# Computed Tomography Perfusion in Suspected Ischemic Stroke: Patient-Related Pitfalls and Practical Clues Beyond Automated Interpretation

**DOI:** 10.3390/brainsci16101079

**Published:** 2026-10-08

**Authors:** Marialuisa Zedde, Giovanni Merlino, Piergiorgio Lochner, Francesca Romana Pezzella, Stefania Miniello, Vincenzo Andreone, Celeste Tucci, Takahiro Ota, Giuseppe Catapano, Vincenzo D’Agostino, Daniele Giuseppe Romano, Luigi Cirillo, Mario Muto, Leonardo Pantoni, Paola Santalucia, Rosario Pascarella

**Affiliations:** 1 Neurology Unit, Stroke Unit, AUSL-IRCCS di Reggio Emilia, 42123 Reggio Emilia, Italy; 2 Neurology Unit and Stroke Unit, Ospedale del Mare, ASL Napoli1 Centro, 80147 Napoli, Italy; 3 SOSD Stroke Unit, Udine University Hospital, 33100 Udine, Italy; 4 Department of Neurology, Saarland University Medical Center, 66421 Homburg, Germany; 5 Neurology Unit, Ospedale Santa Maria Goretti, ASL di Latina, 04100 Latina, Italy; 6 Department of Neurology and Stroke Unit, AORN Sant’Anna E San Sebastiano, 81100 Caserta, Italy; 7 Neurology Unit and Stroke Unit, AORN Antonio Cardarelli, Via Antonio Cardarelli, 9, 80131 Napoli, Italy; 8 Department of Neuroscience, Pediatric Neurology, Santobono-Pausilipon Children’s Hospital, 80121 Naples, Italy; 9 Department of Neurosurgery, Tokyo Metropolitan Tama Medical Center, Fuchu, Tokyo 183-8524, Japan; 10 Neurosurgery Unit, Ospedale del Mare, ASL Napoli1 Centro, 80147 Napoli, Italy; 11 Neuroradiology Unit, Ospedale del Mare, ASL Napoli1 Centro, 80147 Napoli, Italy; 12 Neurordiology, University Hospital ‘San Giovanni di Dio e Ruggi d’Aragona’, 84131 Salerno, Italy; 13 Neuroradiology Unit, IRCCS Istituto delle Scienze Neurologiche di Bologna, 40139 Bologna, Italy; 14 Neuroradiology Unit, AORN Antonio Cardarelli, Via Antonio Cardarelli, 9, 80131 Napoli, Italy; 15 Neuroscience Research Center, Department of Biomedical and Clinical Sciences, University of Milan, 20122 Milan, Italy; 16 Department of Neurorehabilitation Sciences, Casa di Cura Igea, 20129 Milan, Italy; 17 National Centre for Clinical Governance and Excellence of Care, Italian National Institute of Health, 00161 Rome, Italy; 18 Neuroradiology Unit, Ospedale Santa Maria della Misericordia, AULSS5 Polesana, 45100 Rovigo, Italy

**Keywords:** acute stroke, CT perfusion, CTP, ischemic core, penumbra, perfusion mismatch, automated post processing, diagnostic pitfalls

## Abstract

The management of suspected ischemic stroke has evolved significantly, largely due to advancements in imaging technologies such as Computed Tomography Perfusion (CTP). This narrative review aims to delineate the practical applications and limitations of CTP in acute stroke evaluation, particularly in challenging scenarios. It is organized around illustrative clinical and imaging cases regarding small subcortical infarctions, posterior fossa ischemia, abnormal reference hemispheres, chronic steno-occlusive disease, the simultaneous involvement of multiple vascular territories, luxury perfusion, diaschisis, and systemic hemodynamic abnormalities. We discuss the core–penumbra paradigm, emphasizing the need for accurate interpretation of perfusion maps to guide therapeutic decisions. While automated CTP analyses enhance diagnostic efficiency, they can overlook critical patient-specific factors and exhibit variability in performance across different software platforms. Limitations in detecting small infarcts, particularly in lacunar strokes, highlight the necessity for qualitative visual assessments alongside quantitative data. Individualized case evaluations are crucial, as discrepancies between automated readings and qualitative interpretations can influence the management of single cases. Furthermore, we propose an integrated approach that combines automated assessments with human expertise to improve diagnostic precision and treatment efficacy. Future research should focus on refining CTP methodologies and enhancing their applicability in diverse clinical contexts to optimize patient management in acute ischemic stroke situations.

## 1. Introduction

Management of ischemic stroke in the hyperacute phase is currently heavily influenced by the efficacy and wide availability of revascularization therapies, which have driven significant organizational changes in care pathways [1,2,3]. Many decisions regarding patients with suspected acute ischemic stroke are made after a diagnostic evaluation aimed primarily at identifying arterial occlusion, particularly large vessel occlusion (LVO), and assessing the status of the brain parenchyma downstream from the site of occlusion. To achieve these aims, two different pathways are available: a magnetic resonance imaging (MRI)-based and a computed tomography (CT)-based approach [4,5]. The second one is the most commonly employed worldwide, due to the wide availability and accessibility of CT-based techniques. The assessment of brain tissue is primarily based on perfusion techniques, having a role in patient selection and imaging scenarios for the reduced reliability of automated perfusion interpretation also in a subgroup of patients with a low ASPECTS score, although low ASPECTS score and large core categories are partially overlapping but different categories [6]. Perfusion studies rely on the core–penumbra paradigm, which has been recently reevaluated with particular relevance to the concrete possibilities of the current techniques, such as MR perfusion (MRP) and CT perfusion (CTP), to effectively distinguish between the core and the penumbra [7,8]. Perfusion studies have standardized protocols in guidelines and are widely used, particularly through the utilization of automated readings with dedicated software. However, the performance of this software can vary and may not be directly comparable. Among the numerous pitfalls to which the reading and interpretation of CTP in hyperacute stroke studies are subject, some are closely related to patient characteristics and must be recognized to temper the automated reading with that of an experienced human operator, in order to avoid excluding patients who could benefit from therapy, whether intravenous thrombolysis (IVT) or endovascular therapy (EVT). In particular, perfusional evaluation identifying mismatch with predefined thresholds is mandatory for patients presenting in the late time window [1,2,3]. The aim of this review is to illustrate, through clinical scenarios selected due to their challenging features, the potential patient characteristics that may affect the automated software output of the CTP, sometimes leading to misinterpretations, and to describe their pathophysiology.

## 2. Overview of CTP Techniques and Maps for Acute Stroke Evaluation

Perfusion imaging plays a crucial role in assessing cerebral blood flow by utilizing an intravascular contrast agent along with sequential imaging. This technique enhances diagnostic accuracy and helps identify treatment targets while providing valuable prognostic insights into functional outcomes in acute ischemic stroke [4,9]. It is especially beneficial for identifying patients who may be eligible for reperfusion therapy in the late time window or when the onset of symptoms is uncertain [10,11,12].

### 2.1. Techniques and Methodology

Among various imaging techniques, CTP is the most widely used for evaluating acute stroke patients. It is considered particularly effective in automatically assessing core and penumbra mismatch in LVOs, and its widespread use is supported by the current guidelines [1,2,3].

To produce reliable perfusion maps, it is vital to utilize a scanning protocol that maximizes contrast sensitivity while minimizing image noise. This ensures that the bolus passage is adequately captured with high temporal resolution. Balancing these requirements with the need to limit radiation exposure is essential [5].

Post-processing of CTP images involves analyzing a four-dimensional dataset, consisting of three-dimensional volumes captured over time. This dataset can be visualized dynamically, revealing that tissues downstream from an occlusion typically display delayed contrast arrival and prolonged bolus washout [4]. Tracer kinetic models are employed to estimate hemodynamic parameters for each voxel, transforming the four-dimensional dataset into perfusion maps that illustrate various hemodynamic properties. Key parameters assessed include:

- Cerebral blood volume (CBV): The volume fraction of vascularized tissue (typically 2–5%).
- Cerebral Blood Flow (CBF): The volume of blood flow per minute per 100 mL of tissue.
- Mean Transit Time (MTT): The average time for a tracer to traverse the capillary network.
- Time-to-Maximum (Tmax): Indicates the delay of the bolus from the proximal vasculature to the tissue.
- Time to Drain (TTD): Assesses the average duration for a bolus of blood to exit the capillary bed in specific brain regions.

Due to limitations in both CT and MRI, CBF is not typically considered a quantitative measure. Instead, it is normalized to a presumed normal reference region and expressed proportionally. For example, a CBF value of 30% indicates a 70% reduction compared to the reference contralateral region. Other parameters, such as TTP, also exist and can effectively predict infarction when properly normalized to a reference region [13].

A relevant issue is that most perfusion thresholds were derived in LVO patients undergoing EVT with early complete recanalization and reperfusion, and cannot be applied to patients with delayed or partial reperfusion. Usually, at the time of CTP reading, the information about future reperfusion is not available.

### 2.2. Interpretation and Challenges

Visual assessment of perfusion maps can lead to inconsistent interpretations among different readers [14]. To enhance reliability, thresholding techniques are commonly applied to exclude regions with mild hypoperfusion that have a low probability of infarction. However, a limitation of this automated assessment is the challenge of defining correction factors when no normal hemisphere or symmetrical brain region is available for comparison. This situation often arises in patients with prior brain lesions or conditions affecting perfusion unilaterally (e.g., chronic arterial steno-occlusion).

In clinical practice, the primary goals include quantifying two main types of tissue: (1) the penumbra, which is significantly hypoperfused and at risk of infarction without reperfusion, and (2) the ischemic core, which is thought to be irreversibly damaged. By applying specific thresholds, clinicians can estimate the volume of each tissue category, guiding treatment decisions and contributing to clinical protocols. The validation of these thresholds focuses on sensitivity and specificity for predicting the fate of hypoperfused tissue in LVO patients, without considering various influencing factors (e.g., timing of recanalization, systemic conditions) or their applicability in different scenarios [15,16,17].

Numerous software programs are available for generating perfusion maps and estimating ischemic core and at-risk tissue volumes. However, the lack of standardization in perfusion processing results in significant variability across vendors. Comparisons of commercially available software have shown substantial discrepancies in core and penumbra volume calculations, potentially influencing patient selection for reperfusion therapy. Evaluating the performance of such software is beyond the scope of this review, which aims to challenge automated CTP assessments and readings.

Occlusion of a cerebral artery leads to a variable reduction in blood flow within the downstream parenchyma, influenced by the extent of collateral circulation. In regions with inadequate collateral support, a lack of oxygen and glucose can result in cellular energy metabolism failure, leading to infarction [18,19]. While both the ischemic core and penumbra contribute to symptoms, the penumbra remains viable if blood flow is restored [18,19]. Without reperfusion, the penumbra will eventually expand, merging into the ischemic core. Individual rates of infarct progression vary significantly and are heavily dependent on collateral circulation [20]. Genetic and environmental factors contribute to differences in collateral circulation, and stimuli such as chronic hypoperfusion may promote collateral formation [21].

CTP visualizes brain infarction indirectly by assessing changes in perfusion, making small subcortical infarcts more challenging to detect and increasing the likelihood of errors [22,23]. The validation of CTP parameters and thresholds for identifying the ischemic core relies on final infarct volumes determined via follow-up MRI in patients who have undergone complete reperfusion. Variability in optimal parameters can be attributed to differences in imaging acquisition techniques, including brain volume coverage, image acquisition rate, and scan duration, as well as variations in reference imaging and data processing methods, such as arterial input function placement and corrections for delay and dispersion [4,5].

### 2.3. Key Indicators and Thresholds

A decrease in CBF relative to normal brain tissue (rCBF) serves as the most reliable indicator of the ischemic core among the known perfusion parameters, with an rCBF threshold of less than 30% being well validated, although only in patients imaged at specific time points and without very rapid reperfusion. Evaluating perfusion status provides a snapshot aiming to predict outcomes using probabilistic thresholds. These thresholds suggest that rapid recanalization of LVO may help preserve irreversibly damaged brain tissue. However, limited studies using acute MRI to validate ischemic core thresholds indicate that validated CBF thresholds might overestimate the ischemic core when imaging is performed very early after symptom onset [24,25,26]. The ischemic core may also be overestimated if rapid reperfusion is achieved. Interestingly, the rCBF threshold of less than 30% does not seem to overestimate and may even underestimate the final infarct volume in patients undergoing endovascular treatment [25,26,27].

Lacunar or small subcortical infarcts are typically not automatically detected using CTP thresholds. However, visual examination of time-dependent maps (e.g., MTT, TTP, Tmax, and TTD) demonstrates high specificity but moderate sensitivity for identifying these infarcts, with low sensitivity for infratentorial lesions [28].

The validation of CTP thresholds and post-processing techniques has primarily focused on LVO, emphasizing a more dynamic understanding of varying degrees of hypoperfusion rather than a strict dichotomy between core and penumbra. The penumbra is traditionally defined by subtracting the ischemic core volume from the total volume of critically hypoperfused brain tissue. Various perfusion parameters and thresholds have been proposed, with optimal parameters generally validated against final infarct volumes observed on MRI without reperfusion. The choice of deconvolution method and other post-processing algorithms partly accounts for differences in these parameters. While deconvolved perfusion parameters are commonly used, non-deconvolved maps may also yield comparable accuracy [13,29].

Typically, the perfusion deficit is assessed using parameters related to the concentration–time curve’s temporal profile. Tmax, particularly with a delay of more than 6 s, provides a reasonable estimate of final infarction in patients who do not undergo reperfusion [30]. Notably, Tmax may be the most concordant parameter between CTP and MRP [31].

### 2.4. Mismatch Concept

No single perfusion parameter or threshold perfectly encapsulates the perfusion deficit; rather, it reflects the likelihood of infarction in the absence of reperfusion [32]. The risk of infarction escalates with both the severity and duration of hypoperfusion. Short episodes of severe hypoperfusion may not lead to tissue damage, while prolonged hypoperfusion is more likely to result in injury. Late increases in blood flow within irreversibly damaged tissue can occur due to spontaneous recanalization or enhanced perfusion through collateral vessels [33,34]. This ischemic tissue might be discernible on non-contrast CT; however, it may not be recognized as part of the ischemic core on CTP if blood flow exceeds the detection threshold for the core. Therefore, careful examination of non-contrast images for established infarction is crucial, especially in late-presenting patients.

Changes in the apparent diffusion coefficient (ADC) on MRI evolve over time and are influenced by reperfusion status [35]. Consequently, a single ADC threshold may yield varying outcomes for tissue fate based on the timing of symptom onset and subsequent reperfusion.

The severity of hypoperfusion significantly affects tissue fate. Ischemic tissue with limited collateral circulation progresses to infarction more rapidly than better-perfused tissue [36,37,38]. This observation accounts for interindividual variability in infarct growth rates [39]. In addition to Tmax and the hypoperfusion intensity ratio, rCBV is associated with collateral circulation extent and is a predictor of infarct growth [38]. In patients with chronic hypoperfusion, perfusion deficits may be grossly overestimated if Tmax or MMT thresholds are exceeded [39]. Adjusting for delay and dispersion effects can enhance the reliability of perfusion imaging in these patients, particularly in cases of chronic vascular white matter changes [40].

Finally, the mismatch concept serves as a surrogate for the presence of salvageable brain tissue, indicating a notable discrepancy between the perfusion deficit and the ischemic core. The mismatch ratio (MMR) defines this pattern, with various studies refining its criteria. In the DEFUSE study, a target mismatch (TMM) was established to identify patients more likely to benefit from reperfusion, defined by specific MMR and volume thresholds [36]. The DEFUSE 2 study later refined these definitions for patients eligible for EVT [41]. As previously introduced, the current concept of core/penumbra mismatch is best represented by a dynamic continuum of different perfusional measures [7,8]. However, CTP-derived core is always an estimate based on probabilistic thresholds, and it can be overestimated or underestimated.

## 3. Challenging Scenarios

As previously noted, the use of automated software for post-processing CTP source images aims to identify a target mismatch in patients with LVO. However, this approach does not adequately address the broader question of whether hypoperfused or infarcted brain tissue is present within the examined volume. Additionally, various local and systemic hemodynamic conditions can influence rCBV measurements and time-dependent maps. Consequently, there are instances where the automatic identification of hypoperfusion may be inaccurate and must be critically assessed. In such cases, a direct qualitative evaluation of individual maps can be instrumental in identifying the confounding factors and correcting any conclusions drawn from the automated analysis. The following paragraphs will outline some of these scenarios.

### 3.1. When Normal CTP Does Not Mean Absence of Ischemic Lesion

#### 3.1.1. Lacunar Stroke

A recent systematic review [42] investigated the effectiveness of CTP in diagnosing lacunar strokes. This review encompassed 14 studies involving 583 patients, focusing on the diagnostic performance of CTP for identifying lacunar stroke. The mean age of participants ranged from 59.8 to 72 years, with female representation between 32% and 53.1%. The sensitivity of CTP varied significantly, ranging from 0% to 62.5%, while specificity ranged from 20% to 100%. CTP effectiveness is hindered by several technical variances, including the types of scanners used (ranging from 16 to 320 rows), differences in post-processing software, and the varying definitions of lacunar strokes. Infarcts smaller than 20 mm are often undetectable due to CTP limitations in visualizing minor perfusion deficits. While the core–penumbra model is effective for LVO strokes, it is less applicable to lacunar strokes. Additionally, CTP spatial resolution is insufficient for capturing small perfusion deficits. Variability in perfusion maps and post-processing techniques contributes to inconsistent results across studies. The complexity of lacunar stroke, often stemming from small vessel disease, further complicates their identification via CTP. Coexisting white matter disease can also confound CTP results, making accurate perfusion assessment challenging [43].

**Figure 1:**
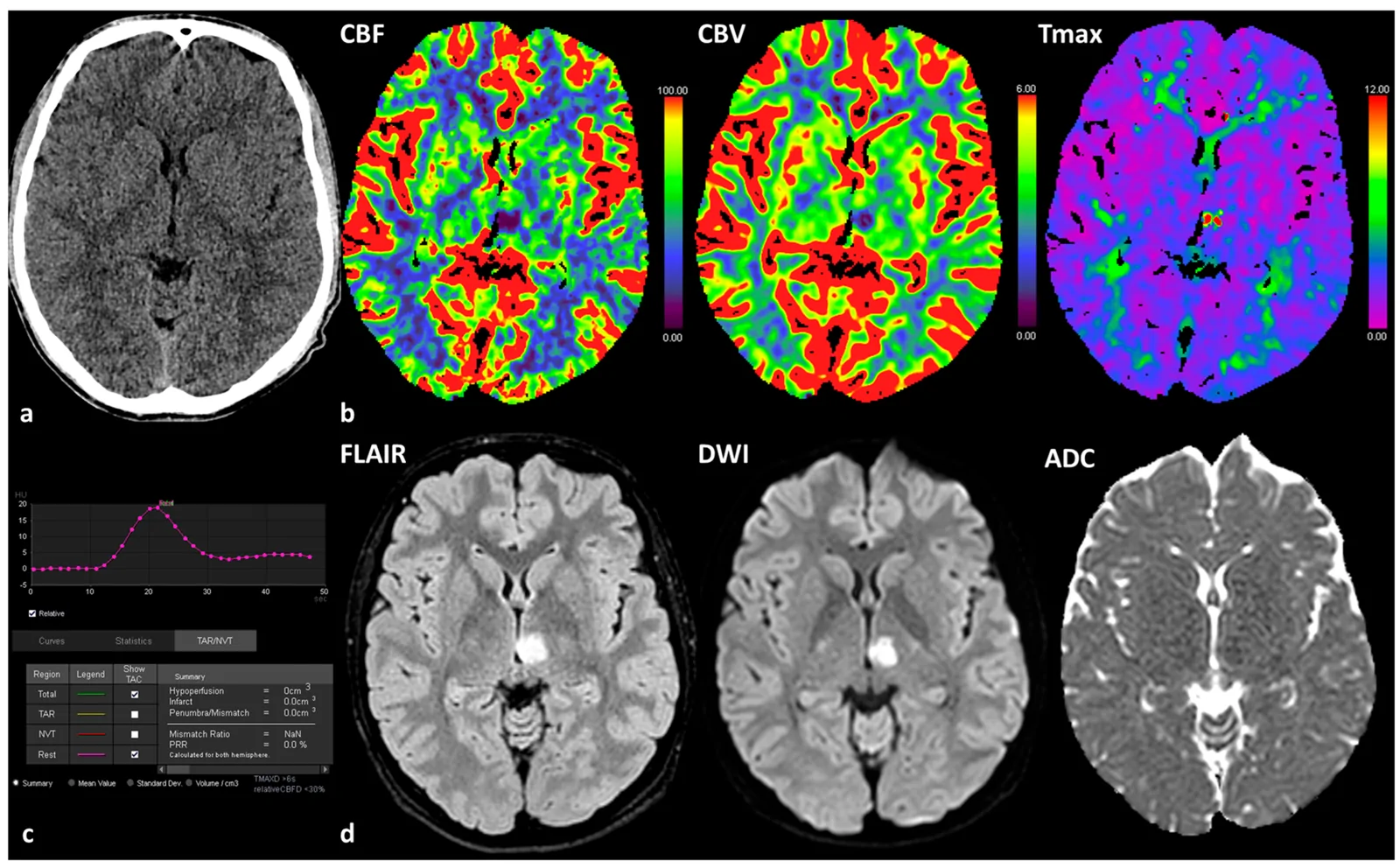
Neuroradiological evaluation of a 26-year-old man admitted to the Emergency Department (ED) because of the sudden onset of right ataxic hemiparesis and drowsiness (LTSW 4 h). Panel (**a**): non-contrast CT (NCCT) at ED admission with normal density of brain parenchyma. Panel (**b**): corresponding CTP maps, showing, on qualitative visual inspection, the presence of a rounded area with low CBF, low CBV and prolonged Tmax in the left medial thalamus, suggestive of acute ischemia and clinically fitting with the patient’s presentation. Panel (**c**): automatic CTP mismatch report, missing the focal hypoperfusion visually identified in CTP maps [panel (**b**)]. Panel (**d**): corresponding MRI at 7 days from admission, confirming the left thalamic ischemia with hyperintense signal on T2-Fluid Attenuated Inversion Recovery (FLAIR) and Diffusion Weighted Imaging (DWI) and pseudo-normalization pattern on Apparent Diffusion Coefficient (ADC) map. CTP was acquired using a 256-row scanner and analyzed using SyngoVia software.

Although CTP may assist in clinical diagnosis in acute settings when a perfusion deficit is observed, its reliability in diagnosing lacunar strokes remains limited. An example is provided in Figure 1.

**Figure 2:**
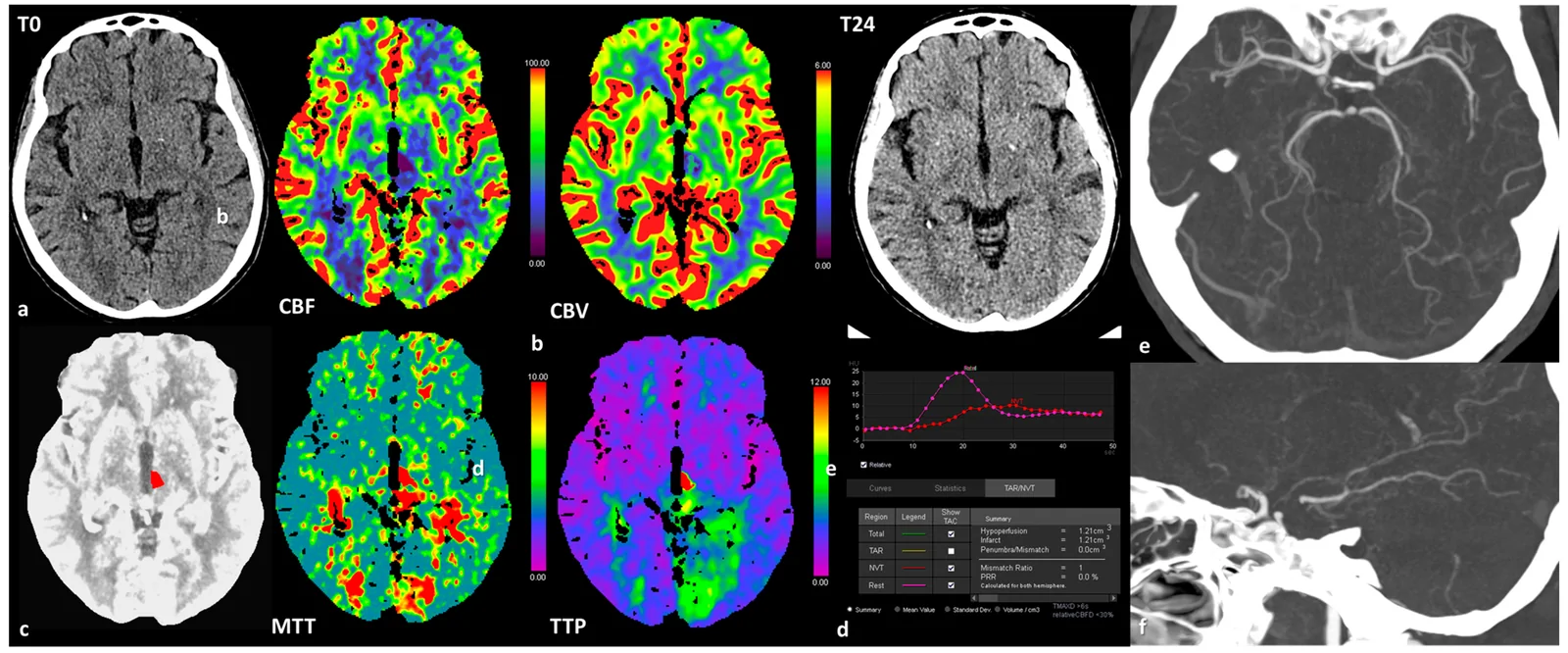
CT evaluation of a 63-year-old man who presented to the ED due to visual disturbances and dizziness (LTSW 8 h). Panel (**a**): NCCT at ED admission, showing the absence of chronic and acute changes in brain tissue density. Panel (**b**): CTP (CBF and CBV in the upper row, MTT and Tmax in the lower row), showing a left thalamic hypoperfusion area in both flow- and time-dependent maps, with a left mesial occipital increase in transit time in the territory of the calcarine branch of the posterior cerebral artery (PCA). Panel (**c**): summary map of the automatic CTP analysis showing a “core” lesion of the left thalamic hypoperfusion, as detailed in panel (**d**) (no mismatch). Panel T24: NCCT after 24 h showing a normal appearance as in panel (**a**), without subacute ischemic lesions. Panel (**e**): CT angiography (CTA), reconstructed according to the Maximum Intensity Projections/MultiPlanar Reconstruction (MIP/MPR) protocol, showing a tight focal stenosis at the P2-P3 bifurcation.

Although the most informative maps for identifying lacunar ischemia are time-dependent maps (TTD and TTP/Tmax), which have the highest sensitivity (49%) and specificity (>97%) [44], CBF and CBV maps may also provide clues, and all maps should be evaluated together. It should be noted that a negative visual inspection does not exclude lacunar ischemia and that sometimes local hemodynamics may affect the CTP appearance and the final fate of the involved brain tissue in a probabilistic approach, as previously remarked and illustrated in Figure 2.

In the case illustrated in Figure 2, CTP maps may reflect an event superimposed on a chronic hypoperfusion disorder with variable degrees of compensation. Therefore, the automatic thresholds used for the maps are not suitable for describing such a situation, resulting in a diagnosis of an ischemic core where no ischemic lesion subsequently developed. An MRI study of the patient is not available because he had insurmountable contraindications to MRI. However, a further NCCT three days later showed no further findings, and the relatively low volume of thalamic lesions may render them below the resolution capabilities of CTP compared to other regions.

#### 3.1.2. Posterior Fossa

The automated core–penumbra CTP maps exhibited decreasing sensitivity for different types of lesions: cortical lesions showed a sensitivity of 56.1%, posterior infarcts had a sensitivity of 25%, and subcortical infarcts demonstrated a sensitivity of only 5.9%. However, these maps maintained high specificity, ranging from 87% to 99% [45]. In contrast, both CBV and CBF maps displayed low sensitivity across all regions (less than 20%) while achieving high specificity (over 95%). Time-dependent maps showed varied sensitivity: low for posterior lesions (less than 30%), moderate for subcortical lesions (between 30% and 40%), and good for cortical lesions (51.5% for MTT). When assessing all CTP maps collectively, the detection rate of subcortical lesions improved significantly compared to the automated core–penumbra maps, increasing from 5.9% to 42.4%. Specificity values remained above 80% for both modalities across all regions [45].

In another case series, increased TTP was observed in 23 patients (47%), and it emerged as a predictor of neurological deterioration in multivariate analysis. Overall, despite technical differences among individual studies, time-based maps were found to be more sensitive than flow-based maps [46,47]. The latter performed better in detecting lesions larger than 20 mm, particularly those caused by the occlusion of multiple perforating branches [48]. The size of the lesion, alongside its location, is critical for detection via CTP, particularly in relation to the average size of the corresponding voxel.

Several technical factors may affect the identification of small hypoperfused regions, although few have been thoroughly investigated. A recent study highlighted the impact of a smaller field of view (FOV) on CTP output for stroke evaluation [49]. With a matrix size of 512 by 512 for brain CT images, the typical FOV in brain CTP is approximately 200–260 mm. The size of the FOV can significantly influence image quality and diagnostic accuracy. The study concluded that higher spatial resolution in CTP images correlates with increased arterial input function (AIF) and venous output function (VOF) peaks, a larger hypoperfusion area, a smaller infarct core region, and a larger mismatch volume [49].

**Figure 3:**
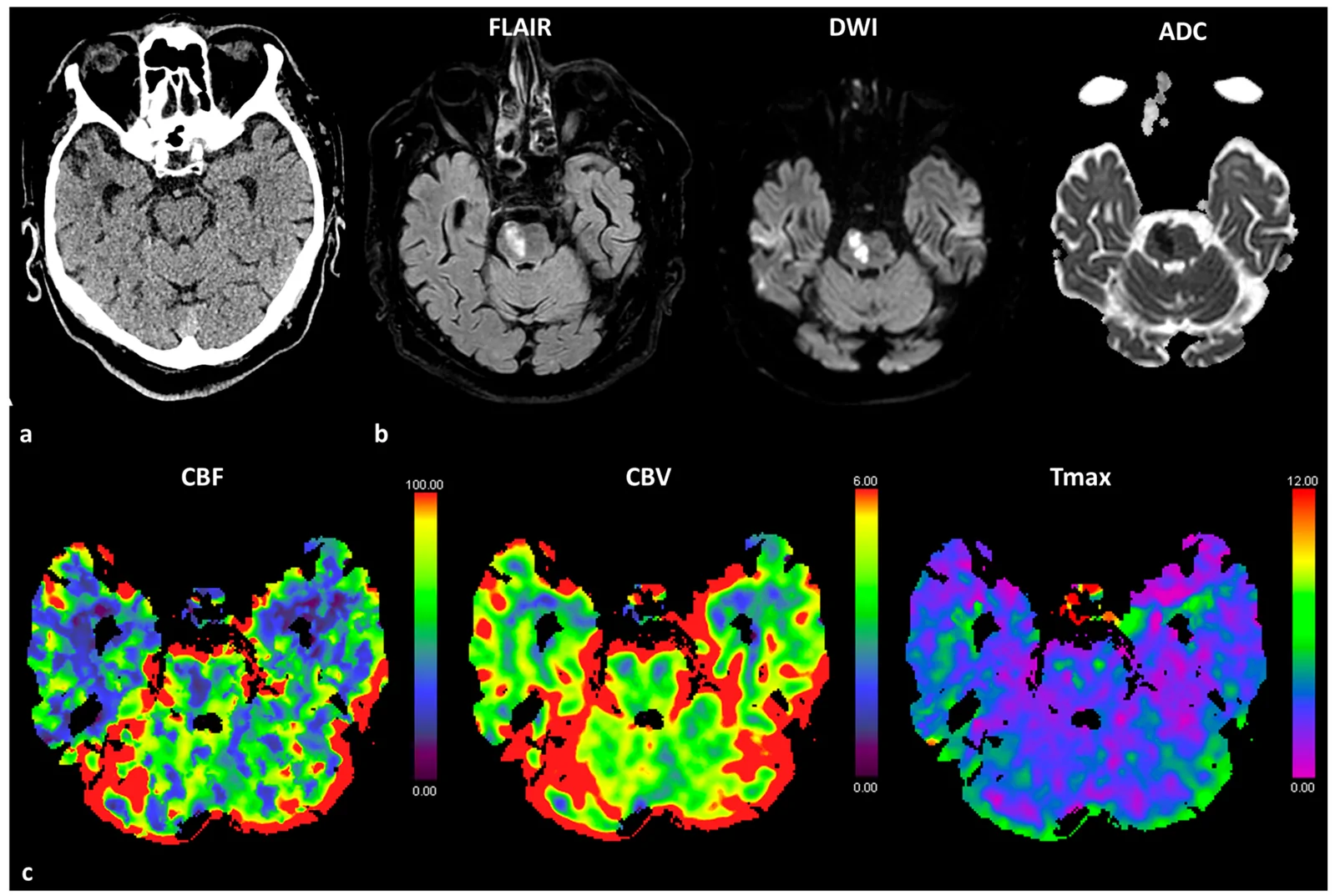
CT- and MRI-based investigations in a patient with basilar artery (BA) occlusion. Panel (**a**): axial NCCT at the lower midbrain level showing a spontaneous hyperdensity of BA without parenchymal changes. Panel (**b**): MRI after three days, highlighting an acute ischemic lesion (hyperintense on FLAIR and DWI and hypointense on ADC map) in the territory of the short circumferential midbrain arteries, arising from the occluded BA. Panel (**c**): CTP without clear visualization of midbrain hypoperfusion (very tiny area of decreased CBF in the right portion of the midbrain without correlation in other maps).

Figure 3 proposes an example of a missed posterior fossa lesion by CTP.

**Figure 4:**
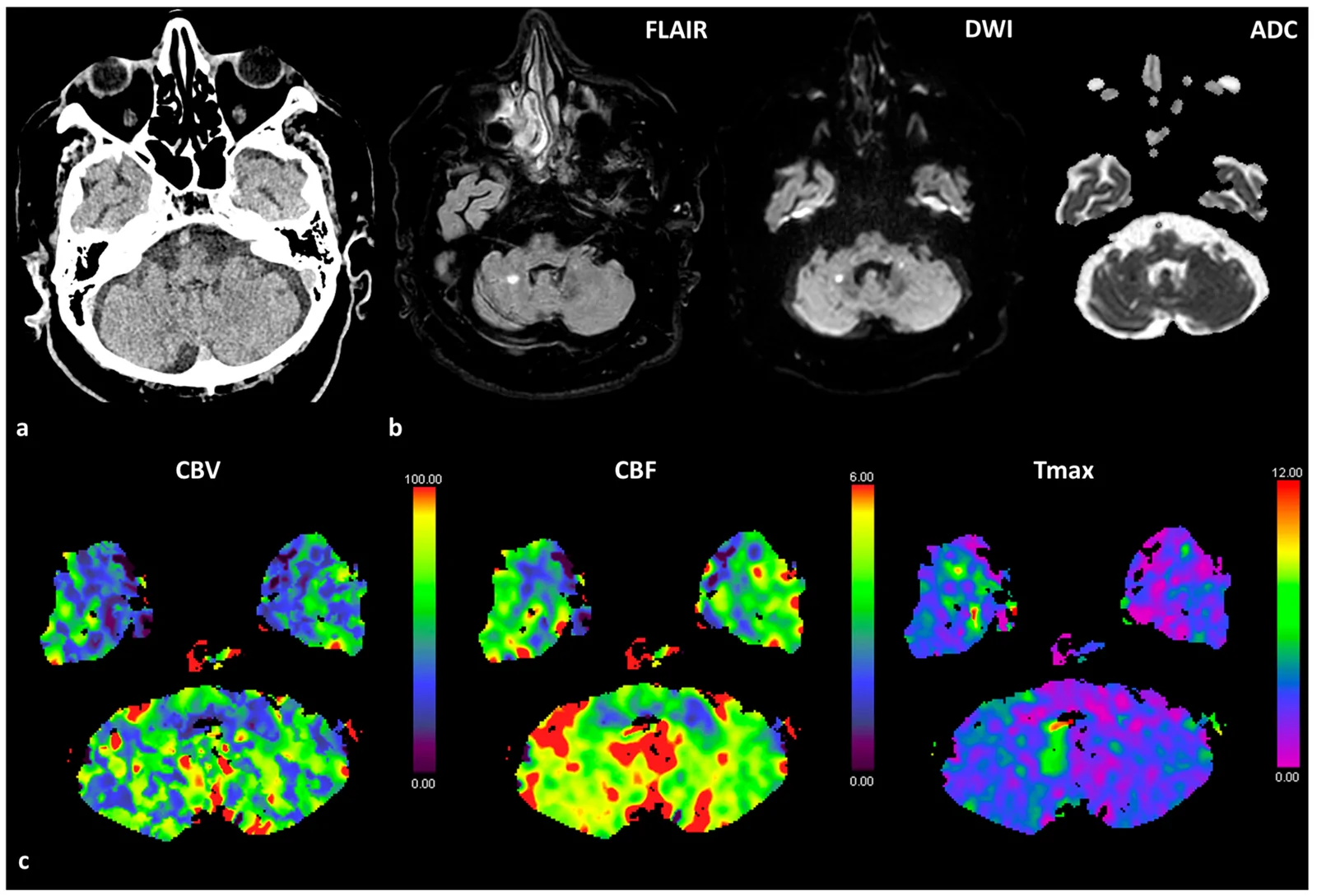
CT and MRI investigation of an 83-year-old woman admitted to the ED for the acute onset of vertigo. Panel (**a**): axial NCCT at the cerebellar level, showing mild chronic changes in the parenchyma. Panel (**b**): MRI performed after 4 days, highlighting a very small subcortical lesion in the white matter of the right cerebellar hemisphere near the middle cerebellar peduncle. Panel (**c**): CTP at the same level, without signs of hypoperfusion in the corresponding area and with Tmax increase in the right paravermian zone.

A similar lack of lesion detection is presented in Figure 4 in the cerebellar region.

**Figure 5:**
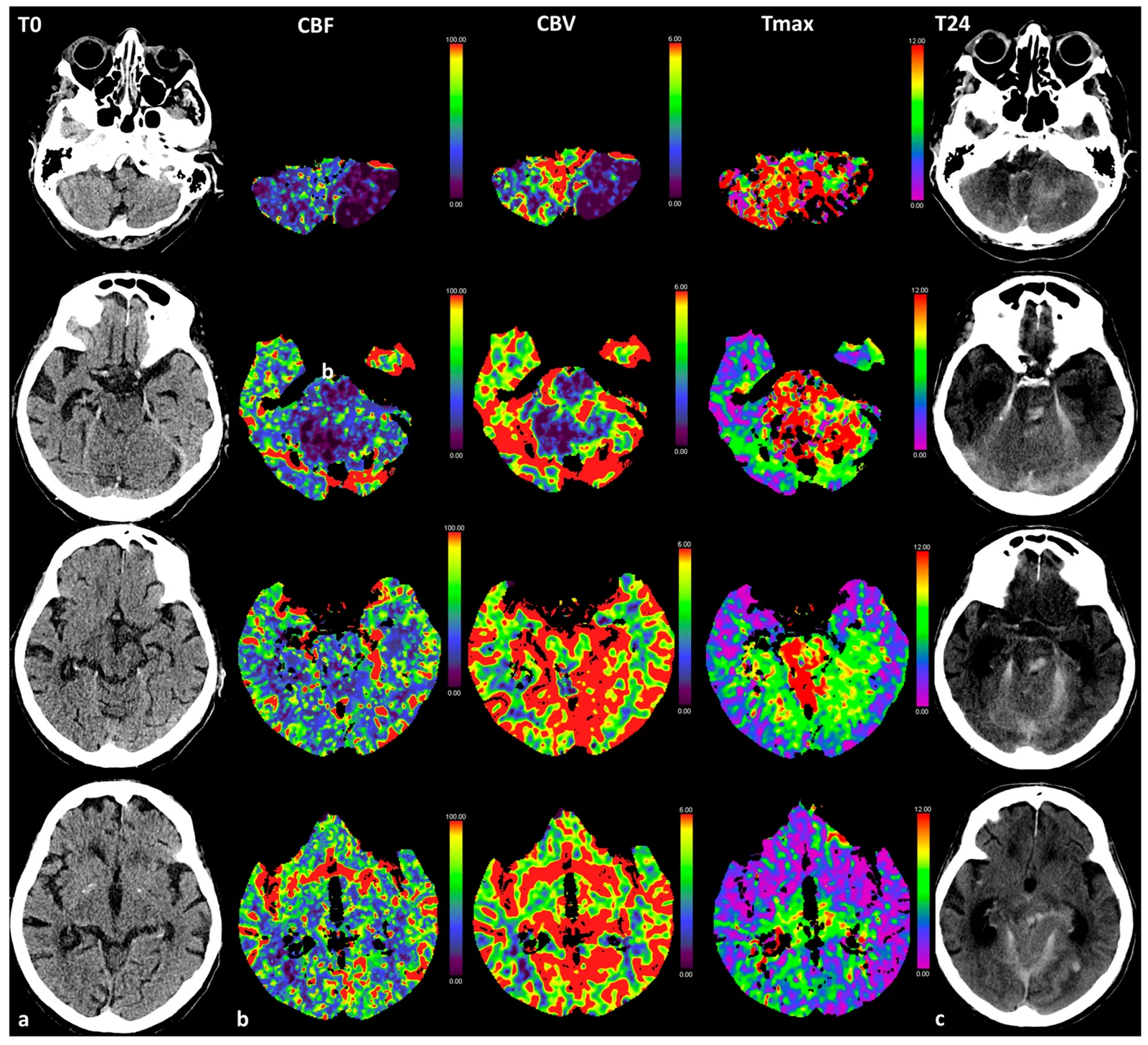
CT-based investigations of an 82-year-old man with a sudden occurrence of coma (GCS 4). Panel (**a**): NCCT at four axial levels (pons, cerebellum, midbrain, and diencephalon in up–down direction), showing an old small right-cortical cerebellar infarction; Panel (**b**): CTP at the corresponding levels, showing a widespread hypoperfusion in the left cerebellar hemisphere, midbrain, right vermis, and PCA territory on both sides in cortical and diencephalic regions. Panel (**c**): NCCT at 24 h after EVT, showing diffuse subarachnoid blood and contrast filling, together with diffuse hypodensity in the hypoperfused regions seen in panel (**b**).

Conversely, Figure 5 shows the usefulness of CTP in posterior fossa lesions when a huge hypoperfusion is present in another case of BA occlusion.

#### 3.1.3. Small Subcortical Infarction

Even in the supratentorial area, the discharge and the location of the ischemic lesion have great relevance regarding the possibility of being identified by the CTP on visual inspection, given that the automated analysis by the dedicated software is even more imprecise in this respect. The main topic is the identification of recent small subcortical infarctions (RSSIs), whose imaging has not been standardized through CTP vs. MRI [50].

The location of restricted DWI signal intensity of RSSI—whether in the internal capsule, external capsule, lentiform nucleus, centrum semiovale, or thalamus—may influence the sensitivity of CTP in identifying these abnormalities. Valdés Hernández et al. [51] reported the volumes of RSSI across various locations during the acute phase: internal capsule/lentiform nucleus (1.23 mL [0.99–1.77]), centrum semiovale (1.32 mL [1.05–1.86]), thalamus (0.51 mL [0.32–0.81]), brainstem (0.72 mL [0.56–0.98]), and optic radiation (1.60 mL [1.51–1.69]). The relatively low volume of thalamic lesions may render them below the resolution capabilities of CTP compared to other regions.

Another important consideration is the relationship between RSSI location and the frequency of identified perfusion abnormalities. An MRI study [52] found that infarction localization significantly correlated with the occurrence of perfusion deficits: 10 out of 13 (76.9%) in the basal ganglia, 15 out of 27 (55.6%) in the internal capsule, 20 out of 27 (74.1%) in the thalamus, 11 out of 34 (32.4%) in the corona radiata, and 3 out of 10 (30.0%) in the brainstem (*p* = 0.003).

**Figure 6:**
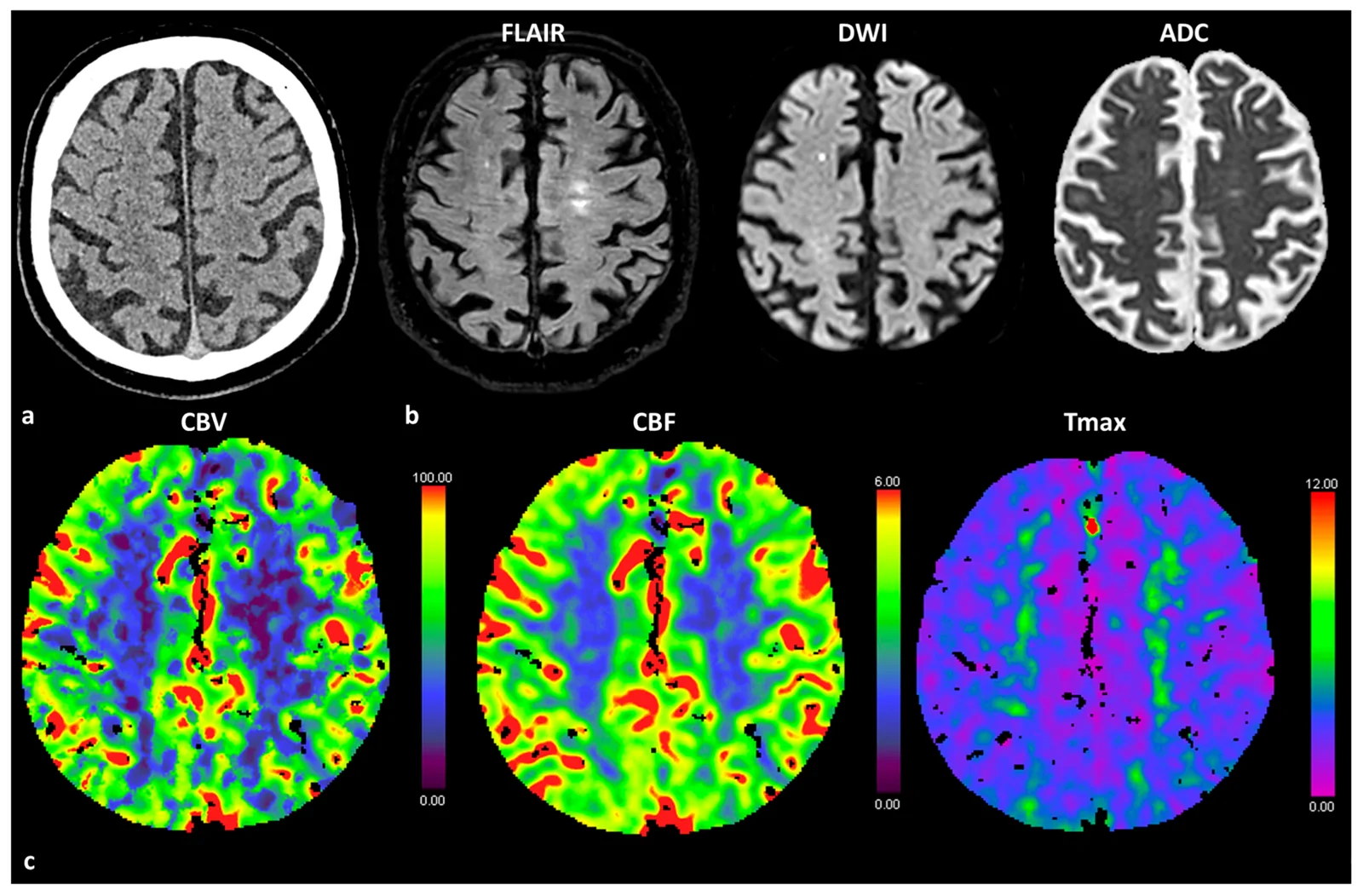
CT and MRI investigations in a 69-year-old man with several vascular risk factors were conducted after he was admitted to the ED due to transient left hemiparesis. Panel (**a**): NCCT at the level of the centrum semiovale shows no significant changes in the density of brain tissue. Panel (**b**): MRI at 4 days after ED admission, showing a small punctate RSSI in the right frontal white matter. Panel (**c**): CTP reveals no evidence of hypoperfusion.

Currently, RSSI does not have a defined lower size boundary, which means the term may also encompass very small, punctate DWI hyperintense lesions in the subcortical white matter. In a study by Tao et al. [53], a retrospective cohort of patients with DWI-diagnosed RSSI was categorized into acute subcortical microinfarctions (diameter < 5 mm) and larger RSSIs (diameter 5–20 mm). They found that 23 out of 584 (3.9%) ischemic lesions belonged to the first group, primarily located in the basal ganglia (11 out of 23), followed by the thalamus (5 out of 23) and centrum semiovale (4 out of 23). An example is illustrated in Figure 6.

**Figure 7:**
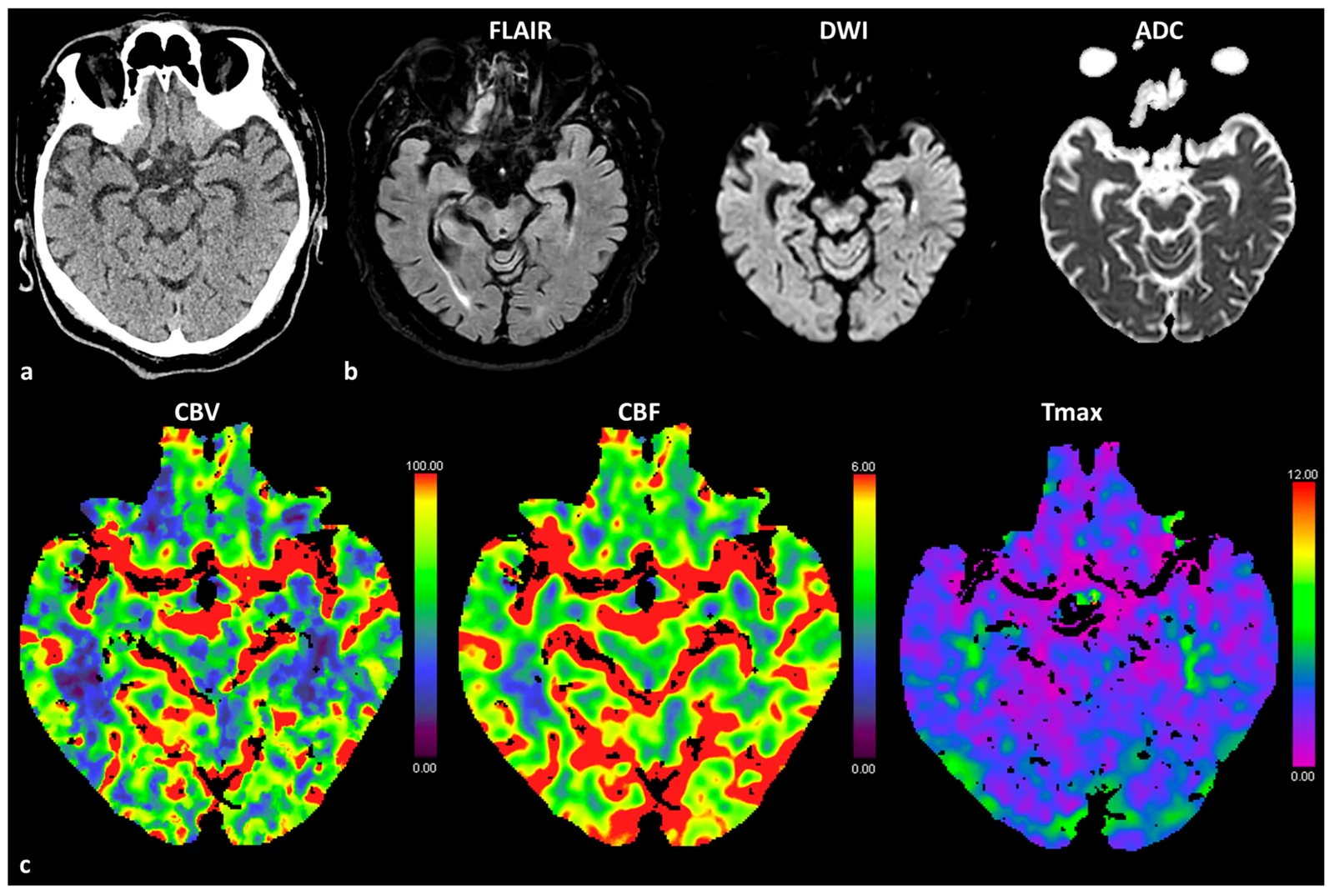
CT and MRI investigations were performed in the same patients as in Figure 6. Panel (**a**): NCCT at the level of the temporal poles shows no significant changes in the density of brain tissue. Panel (**b**): MRI at 4 days after ED admission, showing a small punctate RSSI near the left temporal horn of the lateral ventricle. Panel (**c**): CTP reveals no evidence of hypoperfusion.

In a different location, the CTP performance in identifying RSSIs is the same. In this last case (Figure 7), the unfavorable size matches the unfavorable location near the temporal horn of the lateral ventricle in a patient with multiple RSSIs.

### 3.2. When the Contralateral Reference Hemisphere Is Not “Normal”

**Figure 8:**
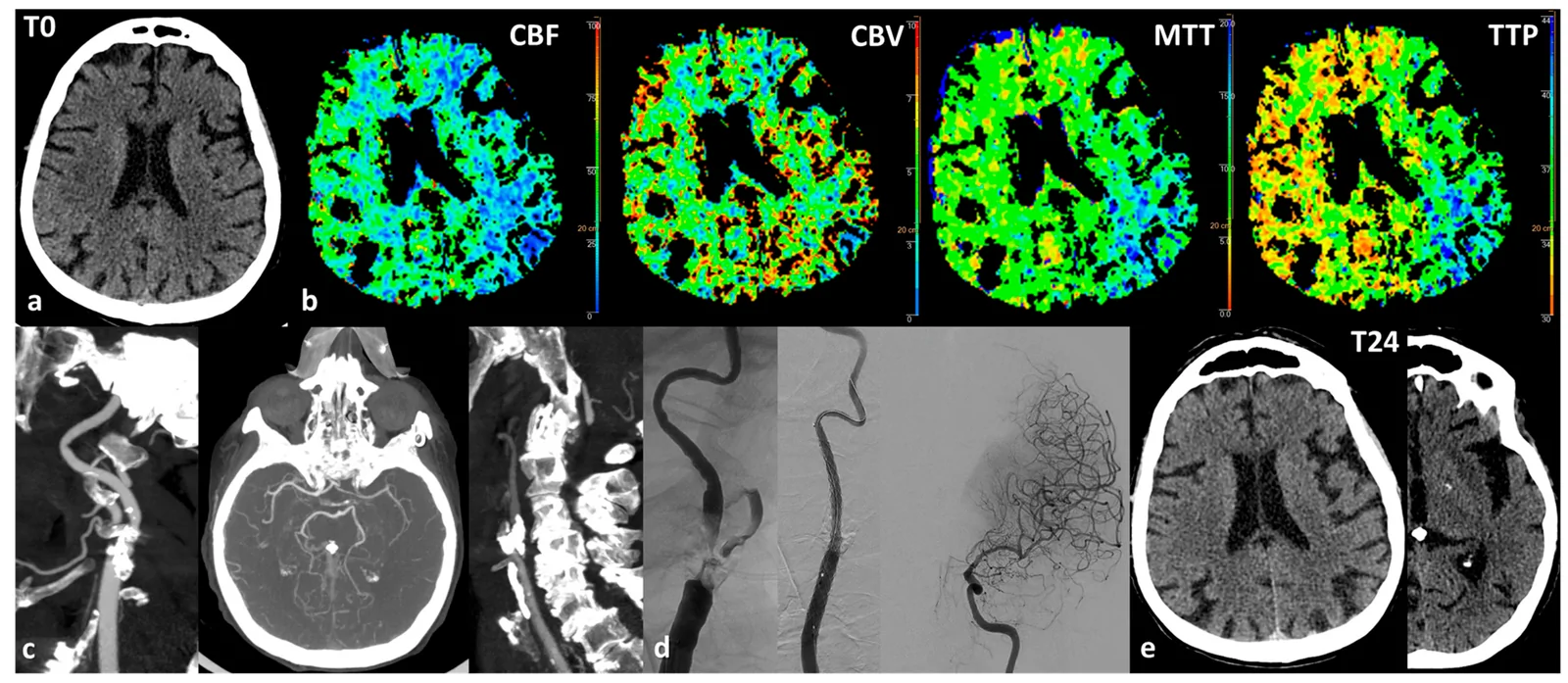
CT-based investigations in a 78-year-old man with sudden onset of aphasia and right hemiparesis (last-time-seen-well–LSTW-5 h). Panel (**a**): baseline NCCT at the ventricular level. Panel (**b**): CTP at the same level, showing a relatively low CBF in the right hemisphere, with lower values in the left hemisphere, a similar CBV, and prolongation of both MTT and TTP in the posterior part of the left hemisphere. Panel (**c**): CTA processed according to MIP/MPR protocol, showing from left to right, the right extracranial ICA with severe stenosis at the extracranial bifurcation, the intracranial supply with a distal left M2 MCA occlusion, and the left ICA with a tight stenosis (near occlusion). Panel (**d**): DSA of the left ICA before stenting, immediately after stenting, and including the MCA (from left to right). Panel (**e**): NCCT at 24 h from admission, showing the lack of ischemic evolution of the hypoperfusion imaged in panel (**b**), with a recent ischemia in the external capsule.

The basis for interpreting CTP maps in post-processing is comparing a region presumed to be pathological (e.g., the hemisphere involved in a LVO) and the corresponding contralateral region, which is assumed to reflect the normal cerebral perfusion of that individual. Unfortunately, this assumption is not always accurate, as various factors—often pre-existing and unknown at the time of evaluation—can influence the perfusion of the so-called normal hemisphere. One such factor is the presence of steno-occlusion of the carotid axis contralateral to a LVO (e.g., a patient symptomatic due to occlusion of the left M1 MCA and with a previously unknown chronic occlusion of the right internal carotid artery). There are not many studies on these issues, and there is insufficient information in the literature to provide correction factors that would reduce bias from overestimation or underestimation related to chronically achieved compensation. Figure 8 illustrates an example of a similar condition.

There is no formally approved strategy to overcome the issue illustrated in Figure 8 in CTP interpretation. Some alternative strategies mainly rely on a qualitative reading of CTP maps, but using absolute thresholds or referring to a different “normal” reference can be postulated.

**Figure 9:**
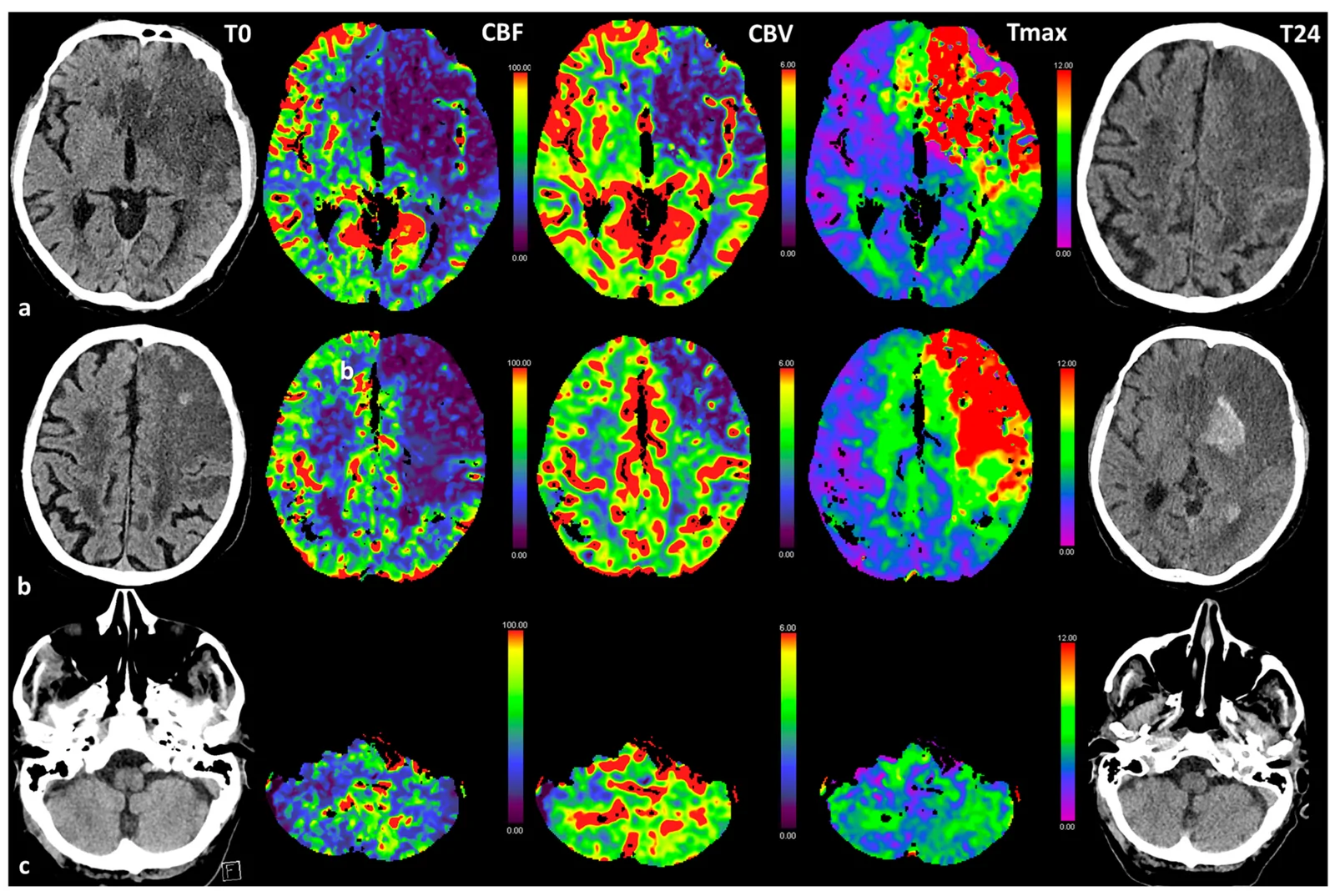
CT-based investigations in a patient with left T occlusion (LTSW 18 h) in an azygos anterior cerebral artery (ACA). Left column: axial NCCT at the diencephalic (panel (**a**)), centrum semiovale (panel (**b**)), and cerebellar (panel (**c**)) levels, showing a hypodense subacute ischemic lesion involving the left MCA territory and ACA territory on both sides. Middle column: CTP, showing the involvement of the same territories with mixed characteristics, with the right ACA territory likely compensated by ipsilateral MCA collateralization. It is interesting to note a bilateral cerebellar hypoperfusion without current or subsequent ischemia, potentially interpretable as an expression of diaschisis (see the next paragraphs for details). Right column: NCCT at 24 h from hospital admission, showing the final fate of the hypoperfused tissue in CTP and deep hemorrhagic transformation; no changes were evident in the cerebellum, supporting the proposed interpretation.

If, in the case presented in Figure 8, the chronically hypoperfused right hemisphere, as a normal reference, may have highlighted the recent left MCA territory involvement, another possibility for having a non-normal reference hemisphere is to have it involved in the acute lesion, as in the case illustrated in Figure 9. In fact, azygos ACA implies that both ACA territories are supplied by the same artery, stemming from ICA. Therefore, when ICA is occluded, hypoperfusion involves both the ipsilateral and contralateral hemisphere to the occlusion site.

### 3.3. When Stroke Is “Strokes”

**Figure 10:**
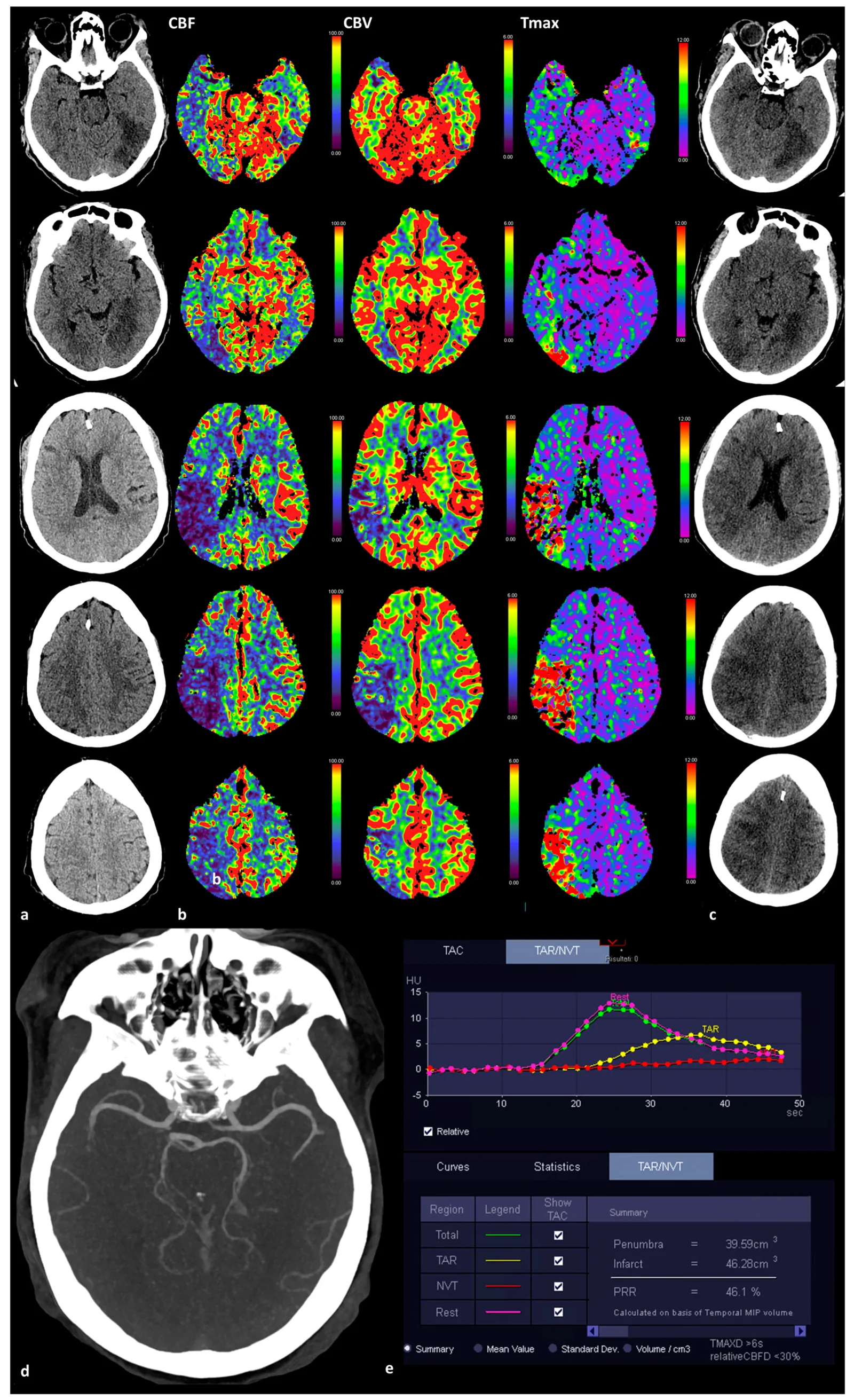
CT-based investigations in a 56-year-old patient admitted to the ED because of right-sided visual disturbances and left hemiparesis upon awakening (LTSW 14 h). Panel (**a**): axial NCCT at pontine, diencephalic, ventricular, and lower and upper centrum semiovale levels (up–down), showing left PCA territory hypodensity (as a subacute ischemic lesion) and hyperacute ischemic signs in the right PCA and MCA territories. Panel (**b**): CTP maps at the same levels, displaying mixed features of core and penumbra in the right lesions and a “perfusion scotoma” in the left PCA territory lesion (probably due to the luxury perfusion phenomenon). Panel (**c**): 24 h NCCT at corresponding axial levels, showing the ischemic evolution of all the hypoperfused areas identified in CTP. Panel (**d**): CTA processed with MIP/MPR protocol, revealing distal P2 PCA occlusion on both sides and right M2 MCA occlusion. Panel (**e**): automatic summary of mismatch provided by the vendor software for the right hemisphere.

Another scenario where automated CTP reading presents difficulties is when there are multiple territorial ischemic lesions, in one or both hemispheres, because the software output is not organized by vascular territory but by hemisphere. Therefore, what is proposed as the core is the sum of the core volumes of the different lesions, and the same applies to the penumbra. In fact, in such cases, the qualitative evaluation of the maps allows for a more reliable identification of whether there is a territory that could potentially benefit from revascularization versus one that is not likely to benefit from the procedure. The presence of multiple lesions also makes it difficult to estimate the clinical correlate of a single lesion, which is why the proposal to estimate a sort of clinical–CTP mismatch on a single lesion also appears less valid. A proposal to distinguish CTP territory according to arterial supply has been made [54], but it has to be proven, and it is not part of routine neurovascular tools. Figure 10 illustrates an example of the above-described scenario, combining in the same patient both luxury perfusion of a subacute infarction and the simultaneous presence of multiple ischemic lesions in different vascular territories. In this case, luxury perfusion is a potential cause of perfusion scotoma (i.e., underestimation of core volume due to the reperfusion of necrotic tissue), but the presence of multiple ischemic lesions is an added reason for the reduced reliability of automated perfusion interpretation.

### 3.4. When Old and New Steno-Occlusion Sum

**Figure 11:**
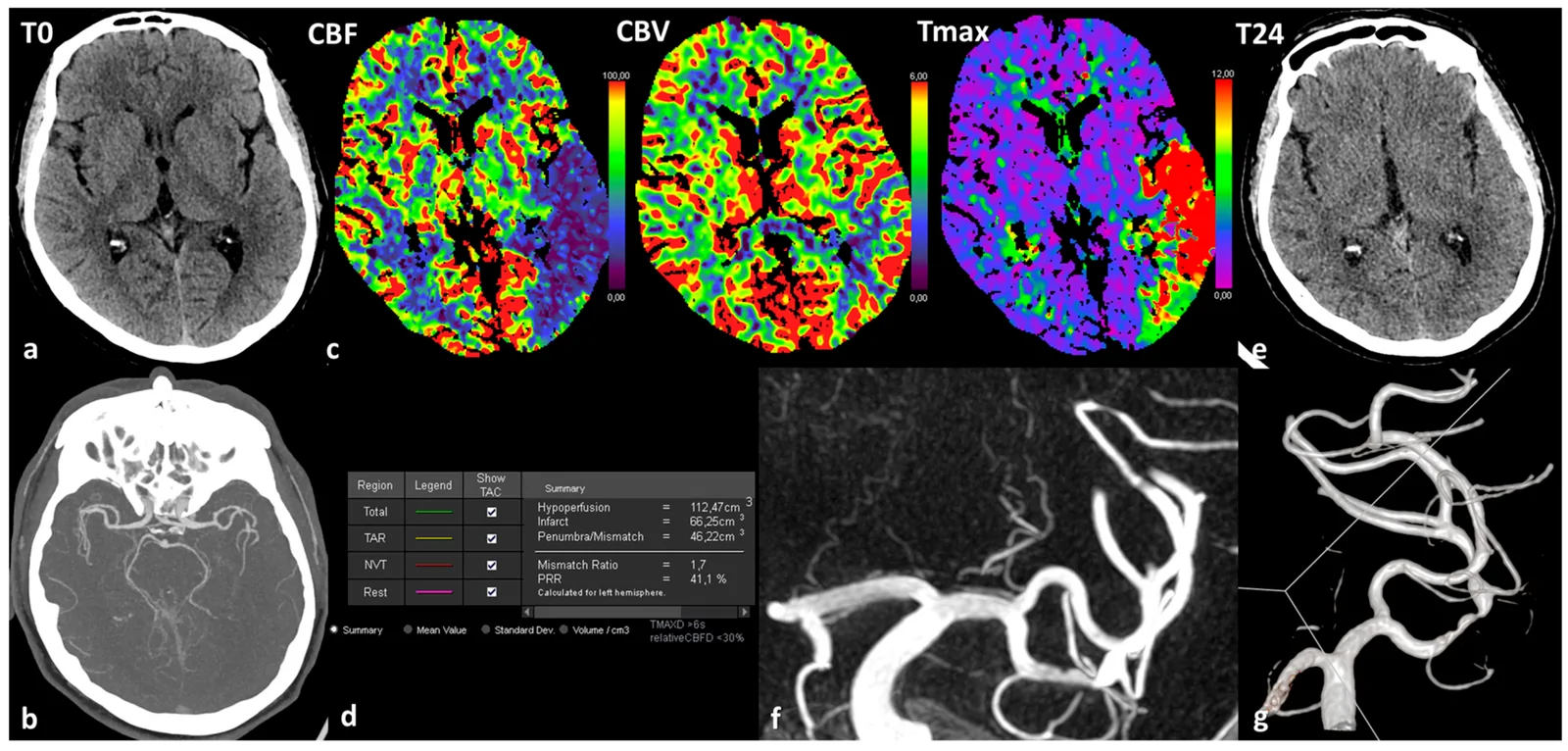
CT-based investigations and DSA of a 76-year-old patient admitted to the ED due to the sudden onset of aphasia and right hemiplegia (NIHSS 22). The time from onset to CT was 90 min. Panel (**a**): NCCT at the basal ganglia level without signal changes. Panel (**b**). CTA processed according to MIP/MPR protocol, showing a focal defect of opacification in the left posterior branch of the M2 MCA. Panel (**c**,**d**): CTP, showing a wide hypoperfused area in the territory supplied by the left posterior branch of the M2 MCA with the main features of core (severely reduced CBF and high Tmax prolongation (see panel (**d**) for summary). Panel (**e**): NCCT at 24 h (no previous IVT and EVT), showing the absence of ischemic lesions and consistent with the spontaneous improvement of the patient (NIHSS 0 at 24 h from admission). Panel (**f**): 3D rotational angiography (RA) reconstructed with MIP/MPR protocol and performed 30 days after CTA, showing partial recanalization of the left posterior branch of M2 MCA with an irregular appearance. Panel (**g**): 3D-RA of the same artery in volume rendering, confirming the irregular luminal surface.

A further scenario that deserves attention is the impact of a new occlusion on an old, even previously unknown, stenosis. This situation could lead, from the point of view of the perfusion maps, to the acute perturbation of a chronic compensation, resulting in a condition that is not clearly readable with the application of the mismatch thresholds validated in conditions of less complexity from the arterial side. An example is provided in Figure 11.

In the proposed scenario, the hypothesis underlying CTP maps and the following clinical and imaging evolution is that the preexisting MCA stenosis may have sustained collateral recruitment, leading to potential chronic perfusion changes, which are then superimposed by a new occlusive event (possibly due to in situ plaque thrombosis). The final CTP maps could reflect the new occlusion on the chronic stenosis, making the CTP thresholds for assessing mismatch less reliable.

### 3.5. When Infarction Is Subacute

**Figure 12:**
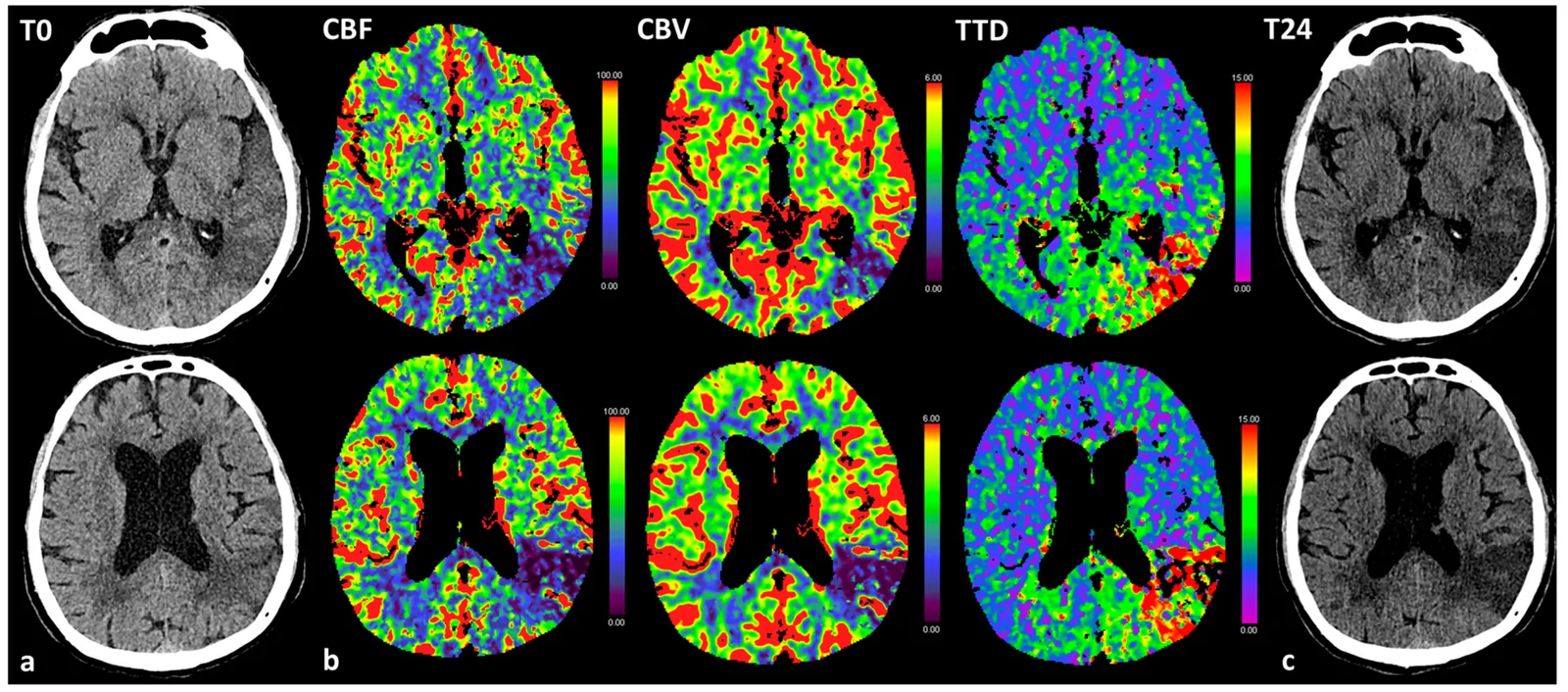
CT-based investigations in a 62-year-old patient admitted to the ED due to right hemiplegia and aphasia upon awakening (LTSW 18 h). Panel (**a**): NCCT at the basal ganglia and ventricular level shows a subacute hypodense ischemia in the left MCA territory involving the temporal and parietal lobes. Panel (**b**): CTP demonstrates “normal” CBF and CBV in the left anterior temporal lobe, with decreased values in the posterior paratrigonal lesion. The TTD map illustrates the differences in the venous phase of blood transit with a normal appearance of the temporal side of the subacute ischemic lesion. Panel (**c**): NCCT at 24 h confirms the hypodense ischemic lesion in the left MCA territory.

Another scenario is that of luxury perfusion, or increased flow in the subacute phase of ischemia, as part of compensatory mechanisms. This phenomenon, described in the literature in various contexts, can also be observed in CTP and may explain the apparent salvageability of brain tissue that is already irreversibly damaged and hypodense in NCCT. Luxury hyperperfusion refers to an excessive blood flow to the brain that surpasses the metabolic and oxygen demands of the tissue. This condition results in non-nutritive flow to the infarcted areas due to an impaired autoregulatory system [55,56,57]. Typically, software algorithms classify luxury perfusion as hyperperfusion on the infarcted side. An example is illustrated in Figure 12, where the volume of tissue with rCBF >30% included late subacute ischemia with defined hypodensity on NCCT and was presented as potentially salvageable tissue even if it is definitely necrotic.

### 3.6. When Stroke Is Not New

**Figure 13:**
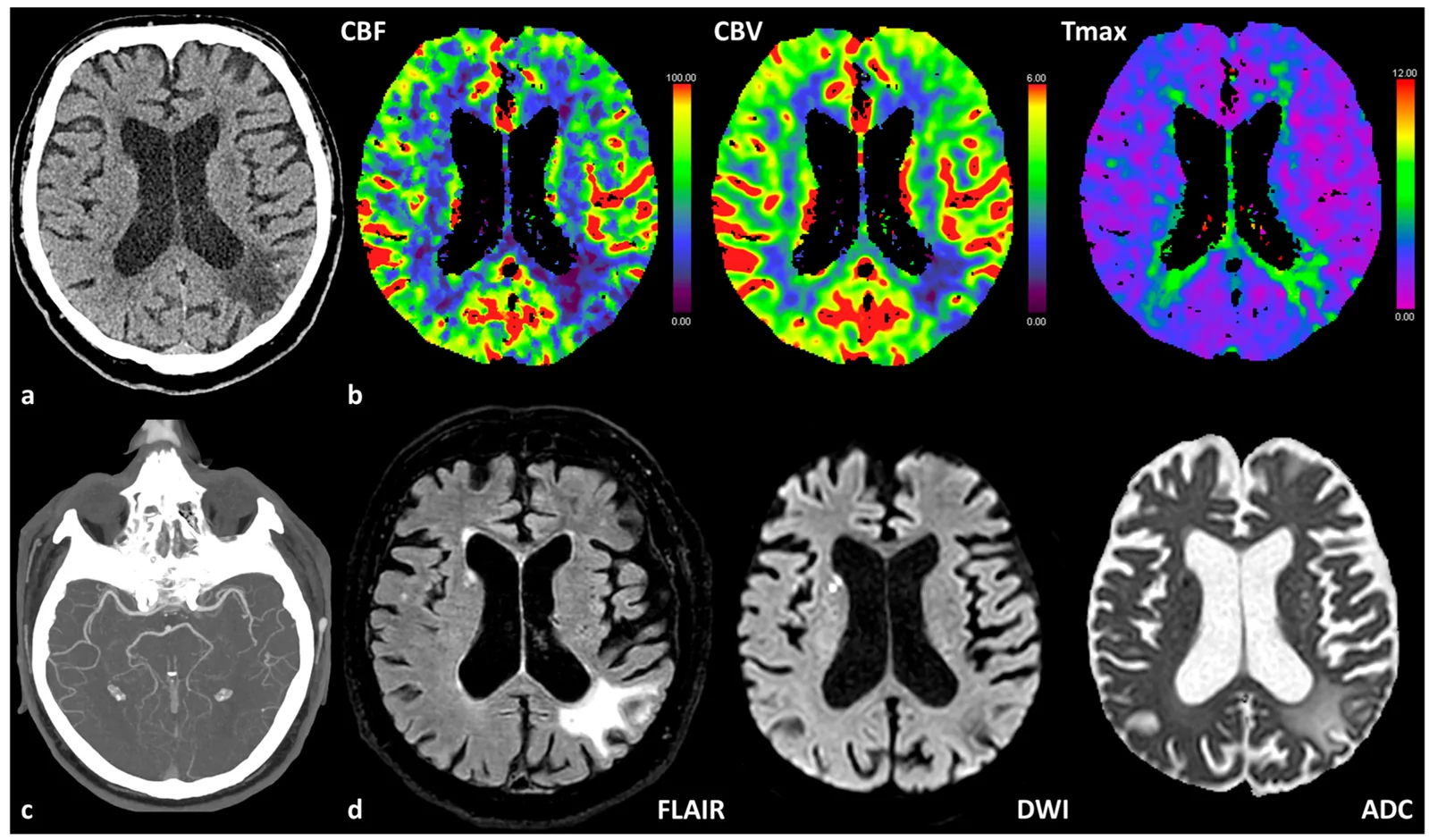
CT- and MRI-based investigations in an 82-year-old man admitted to the ED due to transient left hemiparesis. Panel (**a**): NCCT at baseline shows a hypodense cortical–subcortical old ischemic lesion at the left temporal–parietal–occipital carrefour. Panel (**b**): CTP demonstrates low CBF and CBV, with slightly increased Tmax at the site of the old lesion. Panel (**c**): CTA processed with MIP/MPR protocol shows the absence of LVO. Panel (**d**): MRI reveals an RSSI in the right corona radiata, confirming the chronic timeframe for the left hemispheric lesion.

A similar scenario is represented by the perfusion appearance of an old (not subacute) ischemic lesion, which may interfere with the summary evaluation of the CTP software regarding mismatch both in the affected and reference hemispheres. An example is provided in Figure 13.

### 3.7. When Stroke Is Not Always Only Stroke

In some circumstances, stroke can be the final event of a different disease, as in Posterior Reversible Encephalopathy Syndrome (PRES) with or without Reversible Cerebral Vasoconstriction Syndrome (RCVS) [58,59,60]. It can be posited that similar diseases, characterized by abnormal vascular autoregulation, exhibit a typical finding on CTP studies [61]. Conversely, certain vasomotor phenomena can be consequences of acute ischemic stroke, such as diaschisis [62,63]. The phenomenon of diaschisis refers to a decrease in neural activity, blood perfusion (CBF), or metabolic function in regions that remain intact but are functionally connected to areas affected by a focal brain lesion [63]. Diaschisis has emerged as a crucial concept for understanding the network-level effects of focal injuries. Rather than indicating structural damage, it signifies a secondary functional depression resulting from the disruption of long-range anatomical pathways, including corticocerebellar, thalamocortical, and cortico-subcortical circuits [62]. In the context of stroke, particularly ischemic stroke, phenomena such as transhemispheric, thalamic, and cerebellar diaschisis [63] have been documented during both acute and chronic stages. This remote dysfunction can impact early neurological deficits and subsequent recovery, rendering it a subject of significant interest in clinical neuroscience. Despite its long recognition, the prevalence of diaschisis reported in studies varies considerably. This inconsistency is attributed to differences in patient demographics, timing post-stroke, imaging methodologies, and definitions related to hypoperfusion or hypometabolism. Moreover, diaschisis appears in various forms—including crossed cerebellar, thalamic, cortical, and transhemispheric subtypes—each linked to specific neuroanatomical connections. Among these, crossed cerebellar diaschisis (CCD) is the most frequently noted following hemispheric stroke, typically characterized by reduced perfusion and metabolism in the cerebellar hemisphere opposite to a supralateral infarct [62].

**Figure 14:**
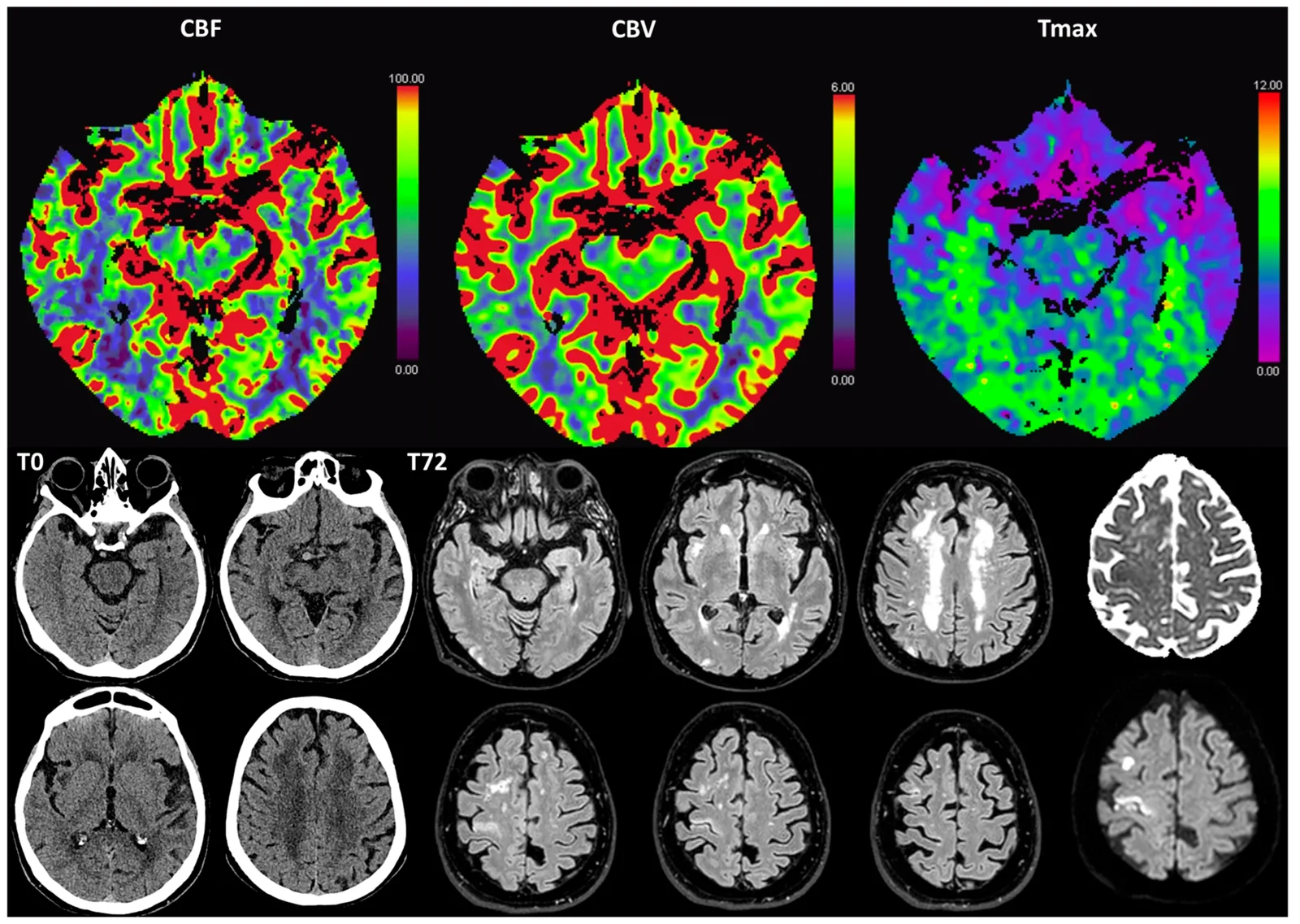
CT- and MRI-based investigations in a 75-year-old woman with a history of poorly controlled arterial hypertension, admitted to the ED due to left arm paresis. LVO was excluded by CTA. CTP panel: posterior bilateral increase in Tmax accompanied by CBV increase in cortical ribbon without evidence of hypoperfusion in any vascular territory. NCCT panel: chronic vascular encephalopathy was imaged, revealing features of small vessel disease without signs of acute lesions. MRI panel: axial T2-FLAIR (six ascending slices) confirming the chronic microvascular changes and the multiple superimposed cortical subacute ischemic lesions, as illustrated in the example in the right column with DWI and ADC map. Due to the acute hospital admission and the initiation of IVT, the patient’s arterial pressure was aggressively managed, reaching the target within 48 h and probably preventing further evolution of the vasomotor phenomenon caught on CTP. It is also possible that some of the white matter hyperintensities highlighted by MRI reflect the superimposition of an acute dynamic change on chronic small vessel disease.

**Figure 15:**
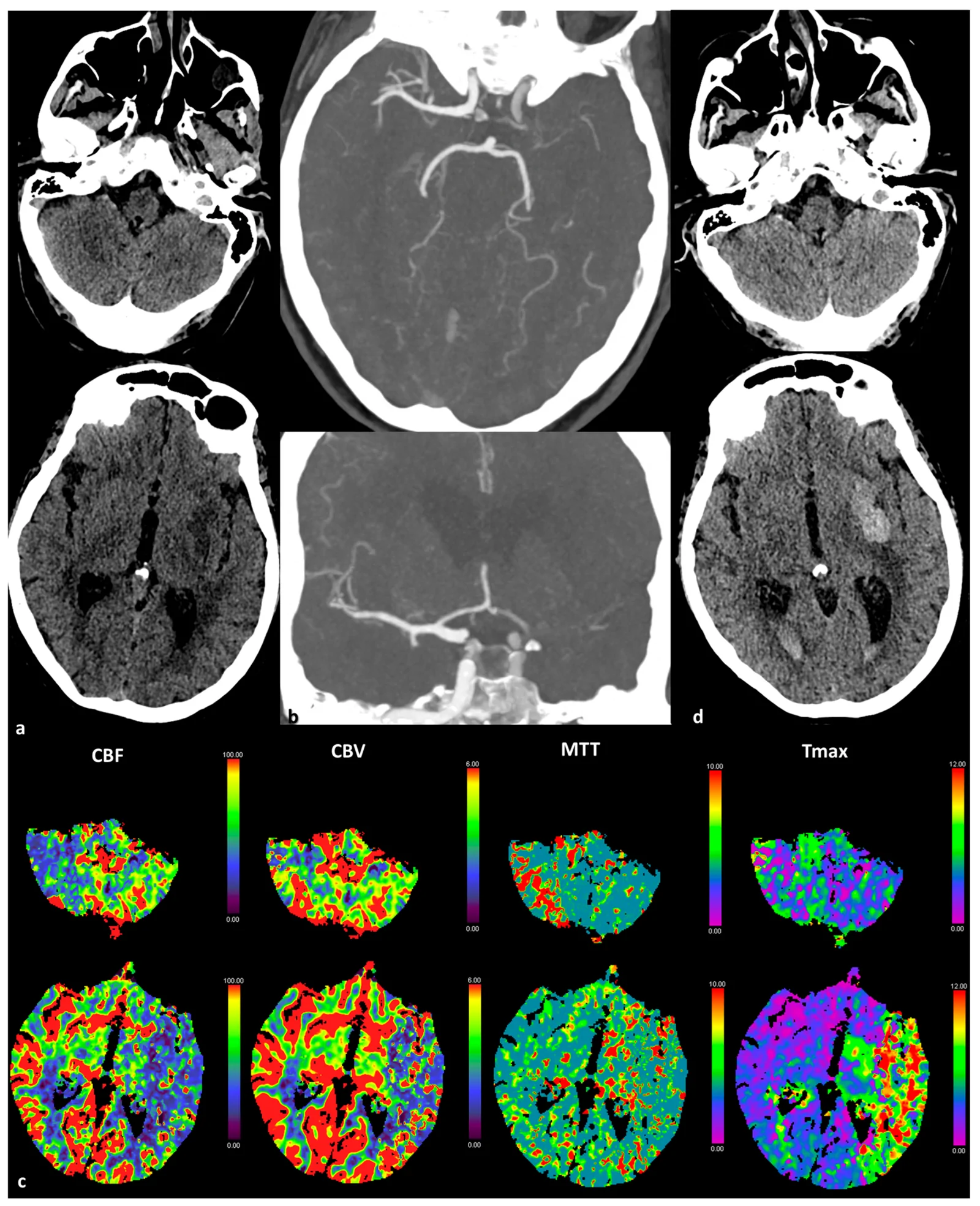
CT-based investigations of a 67-year-old woman admitted to the ED due to right hemiplegia and aphasia upon awakening (LTSW 18 hours). Panel (**a**): NCCT at the level of the cerebellum and basal ganglia reveals the presence of hypodensity in the deep territory of the left MCA. Panel (**b**): CTA reconstructed in axial (upper image) and coronal (lower image) views using MIP/MPR protocol shows left T occlusion. Panel (**c**): CTP at the same levels of NCCT (panel (**a**)) displayed flow- and time-dependent maps. Apart from the large hypoperfusion in the left MCA territory, a decreased CBF with prolonged MTT and Tmax was highlighted in the contralateral cerebellar hemisphere, potentially indicating crossed cerebellar diaschisis (CCD). Panel (**d**): a follow-up NCCT performed 24 h later confirmed the absence of lesions in the right cerebellar hemisphere and showed infarction with hemorrhagic transformation in the left MCA territory (the patient underwent EVT).

Two examples of CTP patterns of these vasomotor phenomena in acute stroke patients are proposed in Figure 14 and Figure 15.

### 3.8. When Blood Goes Slow

One factor that strongly affects any perfusion measurement is the presence of hemodynamically unfavorable systemic conditions at the time of investigation, such as extreme hypotension and severe systolic dysfunction. These conditions prolong the contrast medium distribution curve, affecting the reliability of the measured and calculated parameters compared to patients without alterations in systemic hemodynamics. In fact, low systolic heart function can lead to an overestimation of the core size in CTP, as it slows down contrast arrival and impairs collaterals [64]. Software may misinterpret this delayed contrast transit as an enlarged core, making the affected area appear larger than it actually is. Slower time profiles from scan start to the peak of the arterial input function (AIF) reflect low cardiac output. To mitigate false-positive core sizing in heart failure patients, adjusted, stricter rCBF thresholds may be necessary. Additionally, scans that demonstrate delayed contrast timing often prompt evaluations for low left ventricular ejection fraction. Other potential correction factors include prolonging the acquisition time to capture the venous output phase of the contrast medium, which allows for more reliable application of deconvolution protocols during post-processing.

**Figure 16:**
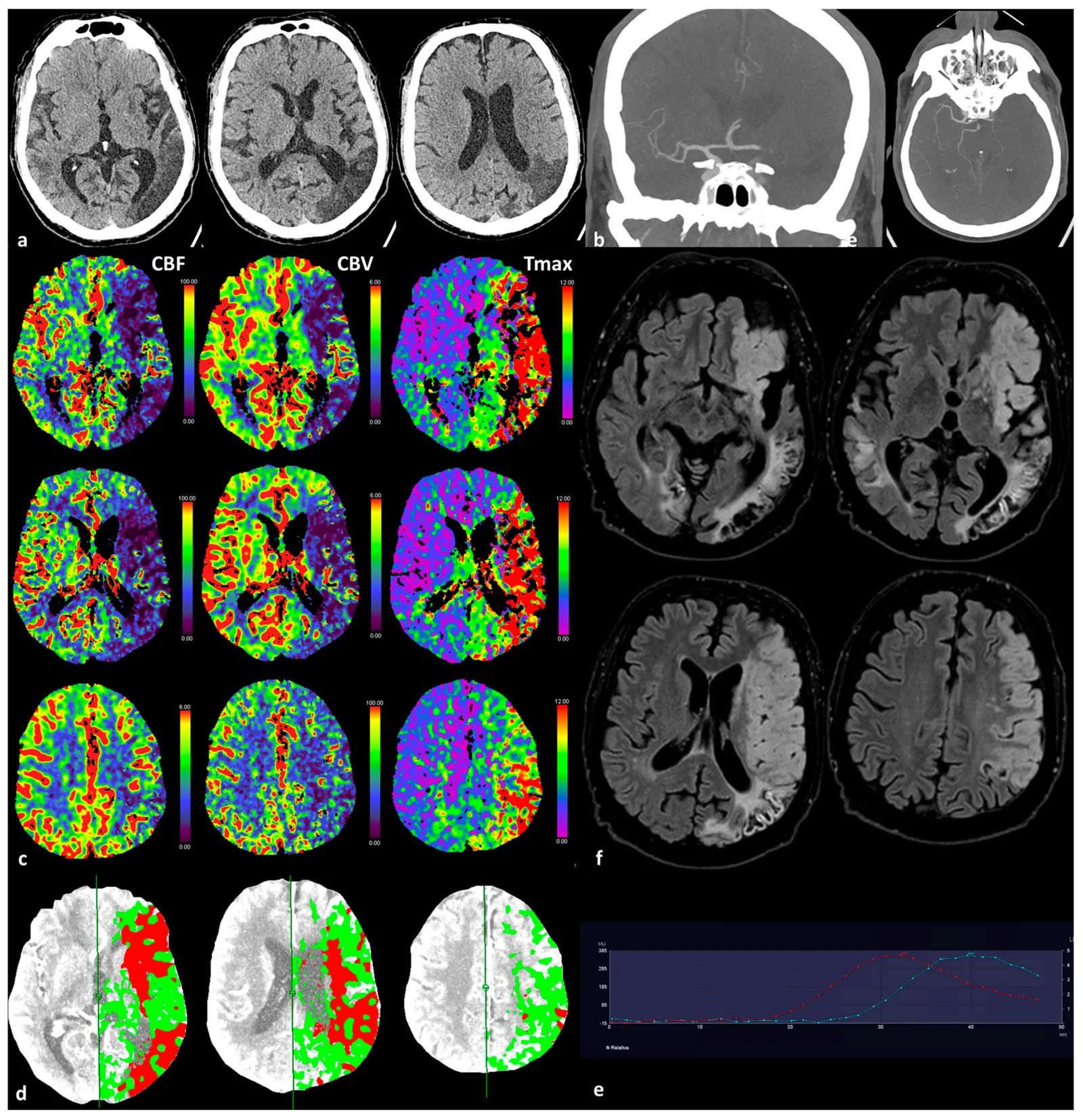
CT- and MRI-based investigations in a 62-year-old man with a systemic amyloidosis secondary to multiple myeloma. He was admitted to the ED due to the sudden worsening of mild right hemiparesis and the onset of aphasia (LTSW 3 h). The patient had a history of previous cardioembolic ischemic strokes and was on anticoagulant therapy. Panel (**a**): NCCT at admission, showing an old infarction in the left posterior MCA territory and a subacute hypodensity in the right posterior temporal lobe. Panel (**b**): CTA processed according to MIP/MPR protocol, revealing a left T type occlusion involving the PCA territory due to a fetal-type PCA pattern. Panel (**c**): CTP showing a large volume of hypoperfused brain in the left hemisphere, comprising both the old lesion and the new occlusion (**d**). The subacute right temporal ischemia must be considered when comparing the ‘affected’ and reference hemispheres by the software. Panel (**e**): Trace of the AIF and Venous Output Function (VOF), demonstrating a marked delay in both, with the AIF not returning to baseline after 50 s. Panel (**f**): Brain MRI, showing some sparing in the left ICA territory previously defined as core by the automatic post-processing software.

An example of this phenomenon is provided in Figure 16.

## 4. Discussion

The automated interpretation of CTP in the context of acute ischemic stroke presents several significant limitations that warrant careful consideration. While automated systems offer efficiency and rapid results, they frequently overlook critical nuances inherent in individual patient cases. This necessitates a more nuanced approach that combines qualitative visual inspection with quantitative data. A summary of the main scenarios and critical issues is presented in Table 1.

The main conclusions about the limitations of automatic CTP reading are the following ones:

1. Limitations of Automated Interpretation
  - Variability in performance: The performance of automated CTP reading software can vary significantly due to differences in imaging protocols, patient anatomy, and underlying conditions. This inconsistency may lead to erroneous assessments of the ischemic core and penumbra, particularly in cases where the perfusion deficit does not align with the expected patterns.
  - Inability to consider individual patient factors: Automated systems often fail to account for patient-specific variables such as previous ischemic events, collateral circulation, and systemic hemodynamic conditions. These factors can alter the interpretation of CTP maps and influence the management of individual cases.
  - Challenges in identifying small or subcortical infarcts: Automated readings often underperform in detecting smaller lesions, such as lacunar strokes, due to limitations in spatial resolution and thresholding techniques. This can result in missed diagnoses and delayed treatment for patients who may benefit from intervention.
2. Importance of Qualitative Visual Inspection
  - Human expertise: Experienced neuroradiologists can provide insights that automated systems may overlook. Visual inspection allows for the identification of atypical perfusion patterns and subtle ischemic changes that automated software might misinterpret or fail to detect altogether.
  - Dynamic understanding of ischemic processes: The qualitative assessment of CTP maps enables clinicians to appreciate the dynamic nature of ischemic processes.
  - Case-by-case reasoning: Individualized reasoning is crucial in complex cases where standard thresholds may not apply. For example, in patients with chronic hypoperfusion or co-existing vascular conditions, relying solely on automated outputs may lead to misleading conclusions regarding tissue viability.
3. Advocacy for Integrated Approaches
  - Combination of quantitative and qualitative methods: To address the limitations of automated CTP readings, an integrated approach that incorporates both quantitative data and qualitative visual assessments is advisable.
  - Future research directions: Further studies should focus on developing hybrid models that integrate automated analysis with expert visual interpretation. Investigating the potential for machine learning algorithms to assist rather than replace human expertise may also yield promising results.

## 5. Conclusions

The use of automated CTP readings in the assessment of ischemic strokes presents opportunities for rapid diagnosis and intervention. However, their limitations underscore the necessity for a more comprehensive approach that includes qualitative visual inspection. Automated CTP output should not be interpreted in isolation, and discordant findings should prompt reassessment of the source maps, NCCT, CTA, vascular anatomy, previous lesions, technical adequacy, and systemic hemodynamic conditions.

## Figures and Tables

**Table 1:** Challenging scenarios in CTP interpretation: mechanism, pitfall of automated interpretation, and practical clues.

Scenario (Figures)	Underlying Mechanism	Pitfall of Automated Reading	Practical Clue	Supporting Evidence
Lacunar stroke (Figure 1 and Figure 2)	Lesion volume below CTP spatial resolution; small vessel disease	Automated core–penumbra maps and mismatch report are unremarkable	Inspect time-dependent maps visually. A negative visual inspection does not exclude lacunar ischemia	Sensitivity 0–62.5%, specificity 20–100% across 14 studies and 583 patients [42]. In 106 clinical lacunar syndromes, median automated core 0 mL and median penumbra 0.4 mL [45] ^a^
Thalamic and basal ganglia location (Figure 1 and Figure 2)	Smallest lesion volumes of all subcortical sites	No detectable abnormality on any map	Do not use CTP to rule out ischemia in these territories; rely on clinical syndrome and MRI	CTP sensitivity 0% in thalami and basal ganglia, versus 21.7–65.2% in subcortical white matter and 12.5–37.5% periventricularly [44] ^b^. Median thalamic RSSI volume 0.51 mL (IQR 0.32–0.81, n = 13) [51]
Posterior fossa, small lesions (Figure 3 and Figure 4)	Beam-hardening artifact at the skull base; small perforator territories	Brainstem and cerebellar lesions not detected	In suspected basilar occlusion a negative CTP has no exclusion value: decide on clinical grounds and CTA	Core–penumbra sensitivity 25% for posterior lesions, AUC 0.66, rising only to 0.69 when delay time and MTT are added [45] ^a^
Posterior fossa, extensive hypoperfusion (Figure 5)	Basilar occlusion with large affected territory	None: CTP is informative	CTP retains value when the hypoperfused volume is large. Its usefulness is a function of lesion size, not only of location	Illustrative case ^f^
Recent small subcortical infarct (Figure 6 and Figure 7)	Unfavorable combination of volume and location; microinfarcts below 5 mm	No perfusion abnormality	Know the expected volumes by site before interpreting a negative study	Median RSSI volumes 0.51 mL thalamus, 0.72 mL brainstem, 1.23 mL capsule–lentiform, 1.32 mL centrum semiovale [51]. Microinfarcts < 5 mm in 23/584 (3.9%) [53]. Perfusion deficit detected in 74.1% of thalamic but only 32.4% of corona radiata lesions (*p* = 0.003) [52] ^c^
Chronically hypoperfused reference hemisphere (Figure 8)	Contralateral chronic carotid steno-occlusion	Normalization against a chronically hypoperfused hemisphere underestimates the acute deficit	Check CTA of both carotid axes before trusting relative thresholds	No validated correction factors available in the literature
Reference hemisphere acutely involved (Figure 9)	Bilateral involvement, e.g., azygos anterior cerebral artery	Interhemispheric comparison loses meaning	Suspect it when symptoms are bilateral or arterial anatomy is variant	Illustrative case ^f^
Multiple territorial lesions (Figure 10)	Simultaneous occlusions in one or both hemispheres	Output is generated per hemisphere, not per territory: core and penumbra are summed across distinct lesions and the mismatch ratio becomes meaningless	Assess each territory separately on visual inspection; the clinical correlate of a single lesion cannot be inferred	Vascular-territory mapping of CTP has been proposed but is not part of routine tools [54]
Acute occlusion superimposed on chronic stenosis (Figure 11)	Chronic collateral recruitment disturbed by a new occlusive event	Thresholds validated in simpler arterial conditions: core overestimated	A large automated core that does not evolve on 24 h NCCT indicates chronic compensation; look for luminal irregularity on CTA or DSA	Illustrative case: NIHSS 22 at 90 min with extensive automated core, NIHSS 0 and no infarct at 24 h without reperfusion therapy
Subacute infarct with luxury perfusion (Figure 12)	Non-nutritive post-reperfusion hyperemia in already infarcted tissue; abolished autoregulation	Classified as hyperperfused or normal: tissue appears salvageable when it is already necrotic	Read the NCCT first. Established hypodensity overrides a reassuring perfusion map	Phenomenon described since 1966 [55]
Chronic infarct (Figure 13)	Cavitated lesion with reduced CBF and CBV	Interferes with the mismatch calculation in both the affected and the reference hemisphere	Identify it on NCCT and exclude the region from quantitative analysis	Illustrative case ^f^
Vasomotor phenomena: PRES and RCVS (Figure 14)	Impaired autoregulation, not occlusion	Tmax prolongation read as ischemia	A perfusion abnormality that does not respect an arterial territory is unlikely to be ischemic	CTP changes described in PRES [61]
Diaschisis (Figure 15)	Remote functional depression through disconnection of long tracts	Contralateral cerebellar hypoperfusion read as posterior fossa ischemia	Cerebellar hypoperfusion contralateral to a supratentorial lesion: suspect crossed cerebellar diaschisis and confirm absence of evolution at 24 h	Pooled prevalence 53% (95% CI 47–58) across 66 studies and 3021 patients; CTP detects it in 49% of cases [63] ^e^
Low cardiac output (Figure 16)	Prolonged contrast distribution curve; delayed arterial input and venous output functions	Slowed transit read as core: overestimation	Inspect the AIF and VOF curves: if the VOF has not returned to baseline by the end of acquisition, the study is unreliable. Check ejection fraction	Left ventricular systolic dysfunction independently associated with core overestimation, aOR 2.52 (95% CI 1.07–5.72, *p* = 0.030); collaterals explain 26% of the effect; an rCBF threshold <25% instead of <35% yields the best correlation with final infarct volume (r = 0.91) [64] ^d^

^a^ Sensitivity values reported in [45] represent the average of two independent stroke neurologist readers, and therefore do not necessarily correspond to whole numbers of patients. ^b^ Brainstem lesions were excluded from the analysis in [44]; the reported 0% sensitivity refers to thalamic and basal ganglia locations only. ^c^ Data in [52] were obtained with magnetic resonance perfusion-weighted imaging rather than CTP, and they are reported here as an indication of the influence of lesion location on the detectability of a perfusion deficit. ^d^ Perfusion thresholds are software-specific and are not interchangeable between vendors. The values cited here refer to the thresholds used in the corresponding source studies. ^e^ The pooled prevalence of 53% in [63] refers to all imaging modalities combined; the 49% detection rate is the Figure specific to CTP. ^f^ “Illustrative case” indicates that the scenario is documented by the cases presented in this review rather than by a published series. Abbreviations: AIF, arterial input function; aOR, adjusted odds ratio; AUC, area under the curve; CBF, cerebral blood flow; CBV, cerebral blood volume; CI, confidence interval; CTA, computed tomography angiography; CTP, computed tomography perfusion; DSA, digital subtraction angiography; IQR, interquartile range; MRI, magnetic resonance imaging; MTT, mean transit time; NCCT, non-contrast computed tomography; NIHSS, National Institutes of Health Stroke Scale; PRES, posterior reversible encephalopathy syndrome; rCBF, relative cerebral blood flow; RCVS, reversible cerebral vasoconstriction syndrome; RSSI, recent small subcortical infarct; Tmax, time to maximum; VOF, venous.

## Data Availability

No new data were created or analyzed in this study. Data sharing is not applicable to this article.
